# FKBP51 inhibition by SAFit2 modulates tau pathology and cognitive deficits in PS19 mice

**DOI:** 10.1186/s13195-026-02107-3

**Published:** 2026-06-06

**Authors:** Andrea Contreras-Marciales, Daniela Mezquite-Garcia, Laura A. Verdina, Ahmed Elnahrawy, Tiara Wolf, Jennifer Guergues, Priyanshi Parikh, Elle R. Hunter, Diana Hernandez Acosta, Stanley M. Stevens, Shannon E. Hill, Laura J. Blair

**Affiliations:** 1 Byrd Alzheimer’s Center and Research Institute, 4001 E. Fletcher Ave, Tampa, FL 33613 USA; 2 Department of Molecular Medicine, University of South Florida, 4202 E. Fowler Ave, Tampa, FL USA; 3 Department of Chemistry, University of South Florida, 4202 E. Fowler Ave, Tampa, FL USA; 4 Department of Molecular Biosciences, University of South Florida, 4202 E. Fowler Ave, Tampa, FL USA; 5 Research & Development, James A. Haley Veterans’ Hospital, 13000 Bruce B. Downs Blvd, Tampa, FL USA

**Keywords:** Tau, FK506-binding protein, Transgenic mice, Mass spectrometry, SAFit2, FKBP51

## Abstract

The accumulation of pathogenic tau protein is linked to cognitive decline and neuronal loss in Alzheimer’s disease (AD), with tau oligomers identified as particularly neurotoxic. The 51 kDa FK506-binding protein (FKBP51) stabilizes these toxic tau oligomers and has been identified as a risk factor for several neurodegenerative diseases. FKBP51 levels increase with age and are especially high in AD brains, suggesting its involvement in disease progression. The development of the selective FKBP51 inhibitor, SAFit2, which can cross the blood-brain barrier, has shown promise in reducing stress hormones, improving stress responses, and mitigating protein-related pathologies in other neurodegenerative models. However, the effects of SAFit2 on tauopathies, such as those seen in AD, have not yet been investigated. Here, the effects of the FKBP51-selective inhibitor, SAFit2, were evaluated in PS19 tau transgenic mice. Mice received a 28-day regimen of SAFit2, followed by comprehensive behavioral, neuropathological, and proteomic analyses. SAFit2 demonstrated effective brain penetrance, with sex-dependent pharmacokinetics. Treatment slowed cognitive decline and depressive-like behavior, with pronounced benefits in male PS19 mice, including improved spatial memory and reduced tau oligomer burden. In females, SAFit2 promoted clearance of AT8-positive tau multimers with some benefit to recognition memory. Proteomic profiling revealed distinct molecular signatures underlying these sex-specific responses: males exhibited upregulation of RNA processing and ribosomal proteins, while females showed restoration of calcium signaling and synaptic drivers. Notably, behavioral recovery occurred independently of widespread neuroinflammation reversal. These findings provide the first in vivo evidence that FKBP51 inhibition by SAFit2 induces sex-specific remodeling of the brain proteome. This study also provides further evidence for the therapeutic benefits of targeting FKBP51 for tauopathies.

**Supplementary Information:**

The online version contains supplementary material available at 10.1186/s13195-026-02107-3.

## Introduction

In Alzheimer’s disease (AD), the accumulation of pathogenic tau is closely associated with cognitive impairment and neuronal degeneration [1–3]. Neurotoxicity has been linked to multiple forms of abnormal tau protein [4, 5], but research indicates that oligomeric forms are a main contributor [6–11]. Our prior work identified the 51 kDa FK506-binding protein (FKBP51) as a stabilizer of neurotoxic tau oligomers in vivo [12]. A recent meta-analysis revealed reduced DNA methylation of the gene that encodes FKBP51, *FKBP5*, as a top risk factor across multiple neurodegenerative disorders, including AD, Parkinson’s disease (PD), and Amyotrophic Lateral Sclerosis (ALS) [13]. The levels of FKBP51 increase steadily with aging and are further upregulated in the AD brain, where FKBP51 is found associated with pathological tau [12, 14, 15]. More specifically, elevated FKBP51 protein can be found in excitatory neurons in the AD brain, which correlate with increased vulnerability [16]. Overall, data suggest that higher levels of FKBP51 causes dysfunction through numerous pathways [17–23], so reductions in FKBP51 may be beneficial to providing resilience from tau pathogenesis.

Efforts to generate FKBP51 inhibitors led to the development of the FKBP51-selective ligand, SAFit2, which does not suppress immune activity like its predecessor, FK506 [24–27]. SAFit2 can penetrate the blood-brain barrier up to ~ 16% [24, 28], which has been shown to lower peak corticosterone (CORT) levels [24], improve stress-coping behavior [24, 29], and reduce hypersensitivity to chronic pain in mice [30, 31], as well as prevent stress-induced depressive-like behavior in rats [32]. SAFit2 treatment also lowered mutant huntingtin (HTT) protein levels in two mouse models of HTT pathology [33] and decreased α-synuclein pathology, neurodegeneration, and motor deficits in a mouse model of PD pathology [34]. Despite the prior links to tau, its effects in models of tauopathy remain unexplored.

This study evaluated a 28-day SAFit2 treatment in PS19 tau transgenic and non-transgenic mice. A detailed behavioral, neuropathological, and proteomic analysis was conducted to determine the impact of FKBP51 inhibition on tau accumulation and related deficits.

## Materials and methods

### Animals

All animal studies were conducted according to the National Institutes of Health *Guide for the Care and Use of Laboratory Animals* and were approved by the University of South Florida Institutional Animal Care and Use Committee (IACUC). PS19 mice (Strain #008169, Jackson Laboratory, Bar Harbor, ME) were bred on a C57BL/6J (Strain #000664) background in our in-house colony. All mice were group-housed with a 12-h light/dark cycle and had *ad libitum* access to food and water.

### SAFit2 plasma and brain quantification

Quantification of SAFit2 concentration in plasma and brain was measured using triple quadrupole liquid chromatography-tandem mass spectrometry (LC–MS/MS QqQ). PS19 and non-transgenic (NTg) littermates aged to 8.5 months (*n* = 6/sex/genotype) received three intraperitoneal (i.p.) injections of SAFit2 (10 mg/kg): at 8:00 a.m., 5:00 p.m., and 8:00 a.m. the following day. SAFit2 (AOB6548; AOBIOUS, Gloucester, MA) was prepared as previously described [28]. Briefly, the compound was dissolved in a vehicle consisting of 0.7% ethanol and phosphate-buffered saline (PBS) supplemented with 5% PEG400 (HY-Y0873A; MedChem Express, Monmouth Junction, NJ) and 5% Tween 80 (HY-Y1891; MedChem Express). One hour after the final injection, blood was collected in a tube with 0.5 M ethylenediaminetetraacetic acid (EDTA) via intracardiac puncture and brains were rapidly dissected. After an incubation step on ice for 15 min, blood was centrifuged at 1,500 x g for 15 min at 4 °C to isolate plasma. Both plasma samples and brain tissue (excluding cerebellum) were snap-frozen and stored at -80 °C until subsequent analysis. Brains were weighed, re-suspended in five-fold volume of PBS supplemented with a 1:100 dilution of Protease Inhibitor Cocktail (P8340; Sigma, St. Louis, MO), Phosphatase Inhibitor Cocktail 2 (P5726; Sigma), Phosphatase Inhibitor Cocktail 3 (P0044; Sigma), and phenylmethylsulphonyl fluoride (PMSF; P7626, Sigma), and then homogenized by a Qiagen Tissue LyserLT (85600; Qiagen, Germantown, MD) with two 5-mm stainless-steel beads (69989; Qiagen) at 50 Hz for 2 min. Using Ostro protein precipitation and phospholipid removal plates (186005518; Waters Corporation, Milford, MA), 100 µL of sample was added to each well, followed by 300 µL of LC-MS grade acetonitrile/1% formic acid, which was forcefully pipetted, aspirated 3 times, and then vacuumed to collect the cleared filtrate. Samples were then dried overnight using a SpeedVac and reconstituted in 500 µL LC-MS grade acetonitrile. Analysis was performed on a 6475 Triple Quadrupole LC/MS (Agilent, Santa Clara, CA) coupled with a 1290 Infinity III LC equipped with a JetStream thermal gradient electrospray ionization source. Resolution of chromatography was assessed for SAFit2 on a 2.6 μm Kinetics C18-Polar column (4.6 × 100 mm, Phenomenex, Torrance, CA) with an eluent of 0.1% formic acid: acetonitrile (20:80, v/v), at a 0.8 mL/min flow rate and a 30 °C temperature. The recorded MS/MS ion transitions were 803.2 to 151.0, 384.2 for SAFit2. SAFit2 recovery from samples was 70% ± 4%.

### Chronic SAFit2 treatment in mice

7.5-month-old PS19 male and female mice (*n* = 12/sex/treatment) and NTg littermates (*n* = 11–13/sex/treatment) were randomly assigned to receive twice-daily i.p. injections of either vehicle or SAFit2 (10 mg/kg), prepared as described above, at 8:00 a.m. and 5:00 p.m. for 28 consecutive days. The experimenter was blinded to the genotype and treatment assignments throughout all procedures, including the use of coded injections. After 2 weeks, mice underwent acute stress assay for serum collection to measure CORT. A battery of behavioral tests began on day 21 of treatment. For behavioral testing, animals were acclimated to the testing room for 30–60 min before the start of the task. All testing, except hindlimb clasping, was conducted at least 1 h after the morning injection and was recorded using ANY-maze 7.5 video tracking software (Stoelting Co, Wood Dale, IL). The tests were performed sequentially on separate days in the following order: hindlimb clasping, open field (OF) test, novel object recognition (NOR) test, tail suspension test (TST), and radial arm water maze (RAWM) with reversal. On the final day of treatment, animals were euthanized by Euthasol overdose (Virbac, Westlake, TX) 1 h after the last injection. Sedated mice were transcardially perfused with 0.9% saline solution before collecting the brain. Left hemispheres were fixed with 4% paraformaldehyde (PFA) (158127; MilliporeSigma, Burlington, MA) and stored in DPBS supplemented with 0.05% sodium azide until sectioning. Right hemispheres were further dissected to isolate hippocampus (HPC), anterior cortex (ACx), and posterior cortex (PCx), which were flash frozen and stored at -80 °C for subsequent analyses.

### Corticosterone (CORT) ELISA

One hour after the morning injection on day 14 of SAFit2 treatment, blood samples were drawn from the submandibular or saphenous vein immediately before and 30 min after an acute stressor (10 min of tube restraint). Blood samples were kept at room temperature for 15–30 min before centrifuging at 2000 x g for 15 min at 4 °C to obtain the serum. CORT levels before and after acute stress were measured using an enzyme-linked immunosorbent assay (ELISA) kit (ADI-900-097; Enzo Life Sciences, Farmingdale, NY), according to manufacturer instructions. Serum taken before the stress was diluted 1:50 and serum taken after the stress was diluted 1:100 in sterile PBS.

### Behavioral assessment

#### Hindlimb clasping 

To assess motor deficits, a hindlimb clasping test was performed on day 21 of SAFit2 treatment, immediately prior to the morning i.p. injection. Each mouse was suspended by its tail for 10 s and hindlimb posture was scored on a 0–3 scale [35]. A score of 0 indicated normal splaying of the hindlimbs, whereas a score of 3 was assigned when the hindlimbs were fully retracted and touching the abdomen for more than half of the suspension period.

#### Open Field (OF) and Novel Object Recognition (NOR) testing 

For OF testing, mice were assessed while being allowed to freely explore an open field arena of 40 cm x 40 cm (7001-0067; San Diego Instruments, Inc, San Diego, CA) for 10 min. Total distance traveled, mean speed and time spent in center (% of total time) were evaluated during the 10 min test. Each arena was cleaned with ethanol and allowed to dry before the next animal entered. OF served as the habituation day for NOR testing. The next day, mice were placed into the same chambers and allowed to freely explore for 10 min while being presented with 2 identical objects (familiar objects). On the following day, mice were again placed into the same chambers and allowed to freely explore for 10 min, but with one of the same objects (familiar) from the day before and one new (novel) object different in shape, color, and texture. The pair of objects were randomly and equitably assigned as familiar or novel objects, to avoid bias or preference for one shape or texture. Tests were recorded using ANY-maze software. Time spent with each object was also manually scored by a blinded experimenter to ensure accuracy. Discrimination index was calculated as (time novel object − time familiar object)/(time novel object + time familiar object).

#### Tail Suspension test (TST) 

On day 24 of SAFit2 treatment, to assess depressive-like behavior, mice were suspended by their tails on a hook using adhesive tape. To prevent tail climbing behavior, a lightweight hollow cylinder (approximately 4 cm in length) was placed around the tail prior to suspension, as previously described [36]. Mice were suspended for 6 min, and tests were recorded using ANY-maze software. Latency to first immobility and immobility time during the last 4 min were manually scored from the recorded videos. Immobility was defined as the absence of escape-oriented behavior.

#### Radial-arm Water Maze (RAWM) with reversal 

The RAWM test was performed as previously described [37]. Briefly, the six-arm maze (CUST-7289; San Diego Instruments Inc) was filled with water and contained an escape platform (7001 − 0398, San Diego Instruments Inc) in one arm (the goal arm). The maze was placed in a room with consistent extra-maze visual cues located at the four cardinal points. For each trial, mice were released from a start arm (pseudo-randomly assigned sequence) facing the center of the maze. The protocol consisted of 15 trials per day for three days. During the first two days (acquisition phase), the goal arm’s location was fixed: on day 1, trials 1–12 alternated between a visible and hidden platform, while trials 13–15 used a hidden platform. On day 2, mice underwent the 15 trials with a hidden platform, and on day 3 (reversal phase), the platform was moved to a non-adjacent arm to assess cognitive flexibility (15 hidden trials). The 15 daily trials were grouped into four blocks: three blocks of four trials and a final block of three trials. Each trial had a maximum duration of 60 s. Performance, including latency to find the platform and number of errors, was recorded with ANY-maze software. An error was defined as any of the following: (1) entry into a non-goal arm; (2) entering the goal arm without reaching the platform; or (3) a 15-sec interval where mice remained inactive or failed to transition between maze locations. Trial data within each block were averaged, yielding four data points per mouse per day.

### Western blot

Tissue from the anterior cortex (ACx), posterior cortex (PCx) and hippocampus (HPC) was pulverized into a coarse frozen powder using the MultiSample BioPulverizer (59012 N; BioSpec Products, Bartlesville, OK). To ensure uniform representation across all hippocampal subfields for multiple downstream assays, the intact HPC was pulverized into a homogeneous mixture, from which an approximately 5 mg aliquot was reserved for subsequent proteomics. Total protein lysates were prepared from the cortex and the remaining hippocampal powder by adding 10 µL of chilled 1X RIPA buffer (cat no. R26200-250.0; Research Products International, Mt Prospect, IL) per mg of tissue powder. The buffer was supplemented with a 1:100 dilution of a Protease Inhibitor Cocktail, Phosphatase Inhibitor Cocktail 2, Phosphatase Inhibitor Cocktail 3, and PMSF, as described above. Samples were then homogenized using a TissueLyser LT with two 5-mm stainless-steel beads at 50 Hz for 90 s. Lysates were clarified by centrifugation at 16,000 x g for 15 min at 4 °C, and the resulting supernatant was transferred to a new tube. Protein concentration was determined using a BCA Protein Assay Kit (23227; Thermo Fisher Scientific, Waltham, MA). Protein lysates were prepared for electrophoresis by adding 2x Laemmli sample buffer (161–0737; Bio-Rad). For semi-reducing conditions, β-mercaptoethanol and heat treatment were omitted. Conversely, for fully denaturing conditions, samples were supplemented with β-mercaptoethanol (5% v/v) and bolied at 100 °C for 10 min. Samples were then separated by Any kD Stain-free Protein gels (5678124, Bio-Rad, Hercules, CA) and subsequently transferred onto PVDF membranes (IPVH00010; MilliporeSigma, Burlington, MA). Following the transfer, membranes were blocked for 1 h at room temperature with 7% non-fat dry milk (M17200; Research Products International, Mount Prospect, IL) in Tris-buffered saline containing 0.1% Tween-20 (TBST). Membranes were then incubated overnight at 4 °C with primary antibodies diluted in the blocking buffer. The specific primary antibodies used are detailed in Table 1. HRP-labeled secondaries (rabbit: 4010-05 and mouse: 1020-05; SouthernBiotech; Birmingham, AL) were used to enable chemiluminescent detection with either SuperSignal™ West Pico PLUS Chemiluminescent Substrate (PI34578; Thermo Fisher Scientific) for strong signals or SuperSignal™ West Femto Maximum Sensitivity Substrate (PI34096; Thermo Fisher Scientific) for weaker signals. Target protein signal intensities were normalized to the total protein load of the respective lane using Stain-Free technology (Bio-Rad Laboratories) according to the manufacturer’s recommendations to account for variations in sample loading. Alternatively, for the fully denaturing blots, target signals were normalized to Actin. Blots were imaged on a ChemiDoc Imaging System (12003153; Bio-Rad) and quantified using Image Lab software (v. 6.1, BioRad).

**Table 1:** Description of antibodies used in this study

Target	Antibody	Dilution	Source	Cat. No.
Total Tau	Tau5	1:4000 (WB)	A kind gift from Dr. Nicholas Kanaan at Michigan State University, originally produced by Dr. Lester I. Binder at Northwestern University	RRID: AB_2721194
	Tau Polyclonal Antibody	1:1000 (IF)	Thermo Fisher Scientific (Waltham, MA, USA)	PA5-27287, RRID: AB_2544763
Tau pSer202 + Thr205	AT8	1:1000 (WB/IF)		MN1020B, RRID: AB_223648
Tau pThr231	pT231	1:1000 (WB) 1:500 (IF)	ABclonal (Woburn, MA, USA)	AP0401, RRID: AB_2771607
Actin	β Actin	1:1000 (WB)	Proteintech (Rosemont, IL, USA)	0536-1-AP; RRID: AB_10700003
Oligomeric Tau	TOC1	1:100 (IF)	Novus Biologicals (Centennial, CO, USA)	NBP3-20162, RRID: AB_3608345
FKBP51	Human/Mouse/Rat FKBP51 Antibody	1:100 (IF)	R & D Systems (Minneapolis, MN, USA)	AF4094, RRID: AB_2103136
GFAP	GFAP	1:3000 (IF)	MilliporeSigma (Burlington, MA, USA)	MAB3402B, RRID: AB_10917109
Iba-1	Iba-1	1:1000 (IF)	FUJIFILM Wako Chemicals (Osaka, Japan or Richmond, VA, USA)	019-19741, RRID: AB_839504
Anti-rabbit	Goat anti-Rabbit IgG Alexa Fluor™ 488	1:400 (IF)	Invitrogen (Waltham, MA)	A11008, RRID: AB_143165
	Goat anti-Rabbit IgG Alexa Fluor™ 647			A-21,245, RRID: AB_2535813
	Goat anti-Rabbit IgG Alexa Fluor™ 594			A-11,037, RRID: AB_2534095
Anti-chicken	Goat anti-Chicken IgY Alexa Fluor™ 594			A11042, RRID: AB_2534099
Anti-goat	Donkey anti-Goat IgG Alexa Fluor™ Plus 594			A32758, RRID: AB_2762828

### Immunostaining

For immunostaining, fixed hemispheres were cryoprotected in consecutive sucrose gradient solutions up to 30% in a phosphate buffer and coronal sectioned (25 μm) on a sliding microtome with freezing stage. Three sections per mouse were used to evaluate staining in cortex, dorsal and ventral hippocampus (dHPC and vHPC, respectively). Free floating sections were stained as previously described [12], using the primary and secondary antibodies detailed in Table 1. For the biotinylated antibodies Alexa Fluor 488 or 647 conjugated Streptavidin was used for detection (S21374 or S11223; Invitrogen). Nuclei were counterstained with DAPI (32670; Sigma). Stained tissues were mounted onto charged ColorFrost™ slides (12-550-423, Fisher Scientific) and coverslipped with Prolong Diamond Antifade Mountant (P36961; Molecular Probes, Eugene, OR). The specificity of pT231 antibody was verified using 9-month-old brain tissue sections from hTau and Tau KO mice (*n* = 2/genotype, Strain #005491, Jackson Laboratory) on a C57BL/6J background.

### Confocal microscopy and image analysis

Immunofluorescent staining was imaged using a Zeiss LSM880 AxioObserver laser scanning confocal microscope (Carl Zeiss, Oberkochen, Germany) with a Plan-Apochromat 20x/0.8 M27 objective. Single-plane images were acquired for all regions of interest (ROIs) based on atlas landmarks using consistent laser power and gain settings for each fluorophore to strictly allow for quantitative comparison. ROIs included the prefrontal cortex (Cx), entorhinal cortex (ECx), and the CA1, CA3, and dentate gyrus (DG) subfields of both the dorsal and ventral hippocampus. Image processing and quantification were performed using Fiji (ImageJ v2.16.0/1.54p) software. For analysis, thresholds were applied uniformly across all images within a given dataset to define positive staining. The data are expressed as the percentage of immunoreactive area (% Area fraction) covering the entire field of view, excluding tissue artifacts.

### Mass spectrometry analysis of chronic SAFit2 treatment

Hippocampal tissues (*n* = 6/sex/treatment/genotype) were processed using the iST 96x kit (PreOmics, Planegg, Germany) according to manufacturer instructions. Briefly, the samples were weighed to approximately 5 mg and lysed in 100 µL kit-provided lysis buffer with 50 mg of glass protein extraction beads (C20000021; Diagenode). They were then probe sonicated at 20% amplitude, 6 cycles, 5 s ON/OFF to homogenize the tissue and shear DNA. The samples were clarified at 17,000 x g for 10 min and the resulting protein was quantified using the Pierce 660 nm protein assay reagent (22660; Thermo Fisher Scientific) with added ionic detergent compatibility reagent (22663; Thermo Fisher Scientific). The samples were normalized to 30 µg protein, then digested and cleaned as directed by the kit instructions. The eluted peptides were then dried down using a SpeedVac and stored at 4 °C prior to resuspension.

The dried peptides were resuspended in 0.1% formic acid in H₂O and further diluted 1:10 (approximately 200 ng/µL) prior to injection onto an Aurora Ultimate CSI reversed-phase C18 column (25 cm × 75 μm i.d., 1.7 μm C18, IonOpticks, Fitzroy, Australia) heated at 50 °C using a NanoElute 2 UHPLC in-line with a timsTOF Pro instrument (Bruker, Billerica, MA) equipped with a CaptiveSpray ion source. The mass spectrometer was operated in data-independent acquisition (DIA) mode and eluted over a 90 min gradient (total run time 120 min including prep and wash) in which mobile phase A was composed of 0.1% formic acid in LCMS grade water, and mobile phase B contained 0.1% formic acid in LCMS grade acetonitrile (ACN). Data were acquired by dia-PASEF mode with a cycle time of ~ 1.48 s over an ion mobility range of 0.70–1.40 1/K0 [V·s/cm2] spanning 250–1425 m/z. Linear calibration using ions at 622, 922, and 1222 m/z (Agilent, Santa Clara, CA) was performed on both ion mobility and m/z.

Subsequent raw .d files were searched with DIA-NN (v. 2.2.0) using a predicted library generated from the Mus musculus UniProt database, which also contained the human MAPT sequence (54742 entries, downloaded January 05, 2025), using label-free quantification (LFQ) with match-between-runs (MBR). Employing the –matrix-spec-q 0.01 command, an additional run-specific < 1% FDR filter at the protein level was applied along with the standard global < 1% protein and precursor FDR cutoff. Additionally, mass accuracy and MS1 accuracy were both set to 15 ppm, in addition to the remaining default settings. Differential expression analysis was conducted in MS-DAP (v. 1.2.2) within RStudio where DIA-NN output and FASTA files were input and associated to specific sample metadata [38]. Group contrasts were set for differential abundance analysis (DAA) using the algorithm MSqRob [39]. Minimum detection and quantification filters of at least 5 out of 6 replicates per group for DAA were used. Sample processing batches were specified as a random variable added to the regression model in MSqRob. Additionally, Variance Stabilizing Normalization (VSN) and then Mode Between protein (MBprot) normalization steps were utilized prior to DAA. The MSqRob q-value threshold, set at 0.01 and log2 foldchange threshold, was set to automatically infer through bootstrapping. This filtering basis was used to achieve a statistically robust list of differentially expressed proteins (DEPs labeled as “TRUE” in output), with a secondary (relaxed) threshold of q < 0.05 and 0.26 log2 foldchange threshold to broaden the set of DEPs for downstream bioinformatic analysis (Supplementary Tables 1–2).

### Bioinformatic analysis and data visualization

Enriched proteins were analyzed in Metascape (v3.5.20250701) [40] to generate Circos plots [41] and enrichment heatmaps. Protein-protein interaction enrichment analysis was also performed in Metascape with default parameters, which uses multiple protein interaction databases (STRING (physical only > 0.132), BioGrid (physical only), OmniPath, InWeb_IM) [42–44], and, then, the Molecular Complex Detection (MCODE) algorithm [45] was applied. Protein-protein interaction enrichment analysis data from Metascape were imported into Cytoscape [46] for STRING visualization [47] and log2fold change values were imported manually. Vector graphics were then exported. Schematics were created using Biorender.com.

### Statistical analysis

Statistical analyses were performed using SPSS Statistics, Version 29.0.2 (IBM Corp., Armonk, NY) and graphs were created using GraphPad Prism for Windows (GraphPad Software, Inc., San Diego, CA) version 10.6.1. Data are presented as mean ± standard error of the mean (SEM) with individual values shown for each animal. Data distribution normality was assessed using the Shapiro-Wilk test. For datasets meeting parametric assumptions, comparisons were conducted using Two-way or Three-way analysis of variance (ANOVA) to evaluate the main effects and interactions of Genotype, Sex, and Treatment. Longitudinal behavioral data were analyzed using Repeated Measures ANOVA with time as the within-subject factor. Significant main effects or interactions were followed by Bonferroni post-hoc tests to correct for multiple comparisons. For non-normally distributed data, the non-parametric Kruskal-Wallis test was employed followed by Dunn’s pairwise comparisons with Bonferroni correction for significant results. Statistical significance was defined as *p* < 0.05. Detailed statistics are displayed in supplementary tables for LC-MS/MS (Supplementary Table 3), behavior (Supplementary Table 4), western blots (Supplementary Tables 5, 6, 7) and immunostaining (Supplementary Tables 8–14).

## Results

### Characterization of SAFit2 brain penetrance in 8.5-month-old PS19 and NTg mice

**Fig. 1:**
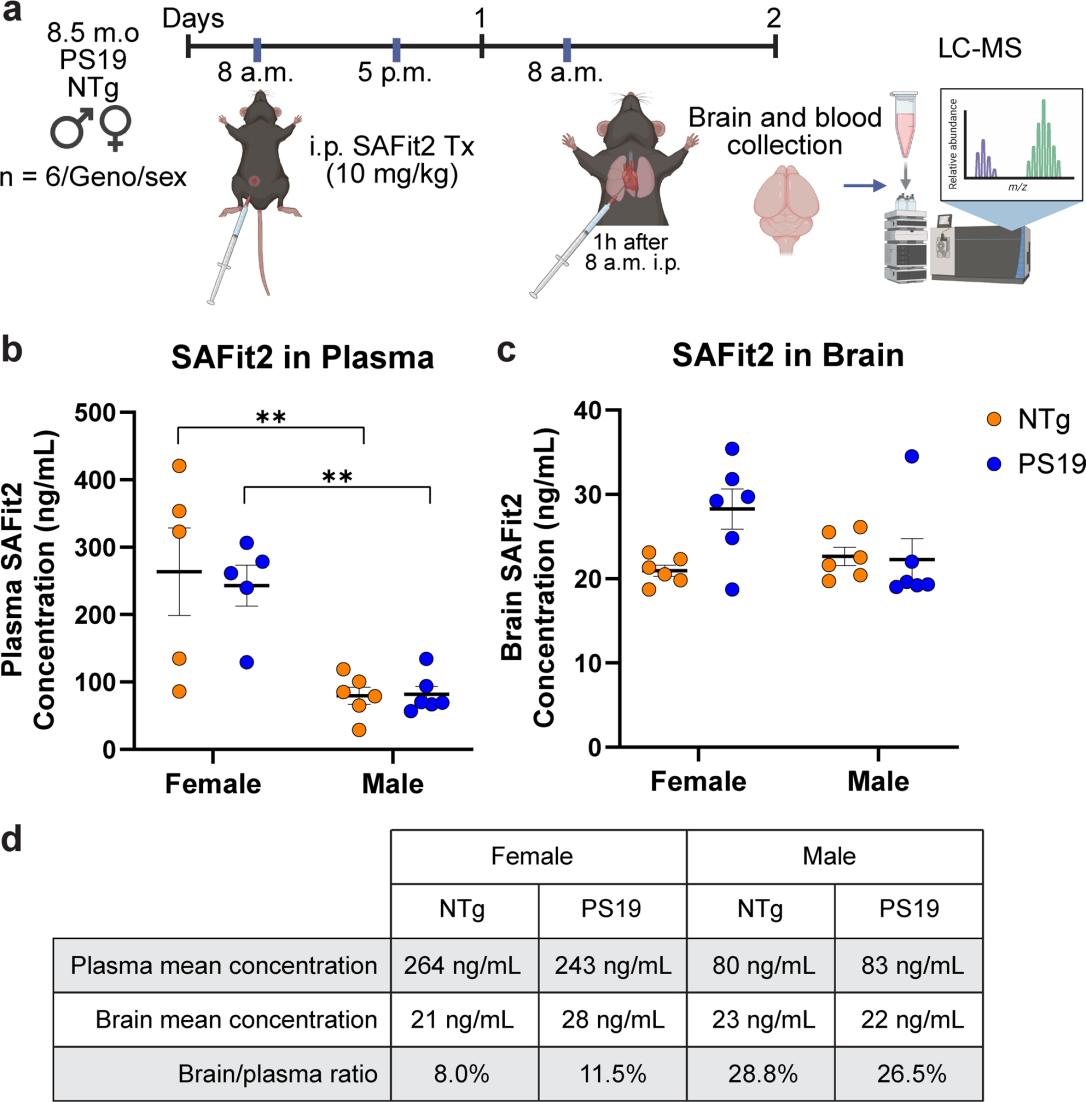
Characterization of SAFit2 concentration in plasma and brain in 8.5-month-old mice.** a** Experimental schematic and timeline. 8.5-month-old male and female non-transgenic (NTg) and PS19 tau transgenic mice (*n* = 6 mice/sex/genotype) received three Intraperitoneal (i.p.) injections of SAFit2 (10 mg/kg) over a 24-hour cycle to reach steady-state levels. Blood and brain tissues were collected 1 h after the final injection for LC-MS analysis. Quantification of SAFit2 concentrations in (**b**) plasma (ng/mL) and (**c**) whole brain homogenates (ng/mL). **d** Summary table of mean concentrations and Brain/Plasma ratios (%). Data are presented as mean ± SEM and n is indicated by individual points. Statistical significance determined by Two-way ANOVA followed by Bonferroni’s post-hoc test (for plasma) and Kruskal-Wallis test followed by Dunn’s multiple comparisons test (for brain). Detailed statistics are provided in Supplementary Table 3, **p < 0.01

To optimize a successful treatment strategy, SAFit2’s brain penetration and plasma concentration were measured. Previous studies with similar dosing (10 mg/kg i.p.) have shown SAFit2 is bioactive [28], but drug pharmacokinetics can vary significantly by age and disease state (Reviewed in [48, 49]). SAFit2 (10 mg/kg) was administered 3 times by i.p. injection into 8.5-month-old PS19 tau transgenic and non-transgenic (NTg) mice (6 mice/genotype/sex) over a 24-hour cycle (8:00 a.m., 5:00 p.m., and 8:00 a.m. the next day). Plasma and brain tissues were collected 1-hour post-final injection for LC-MS analysis (Fig. 1a).

Quantification of plasma levels revealed a significant sex difference. Females exhibited approximately 3-fold higher circulating concentrations compared to males regardless of genotype (NTg *p* = 0.001 and PS19 *p* = 0.003) (Fig. 1b). Although plasma levels were lower in the aged cohort compared to prior reports in younger mice [24, 50], SAFit2 successfully crossed the blood-brain barrier (BBB) in both sexes and genotypes, reaching similar brain concentrations (21–28 ng/mL) (Fig. 1c). Analysis of the brain-to-plasma ratio revealed notable differences in transport efficiencies. Male mice exhibited a more efficient brain uptake (ratio ~ 26–29%), whereas females showed a lower ratio (~ 8–11%) (Fig. 1d). Notably, the measured brain concentrations (~ 25 ng/mL or ~ 30 nM) exceed the inhibition constant (K_i_) of SAFit2 for FKBP51 (~ 6 nM) [50], suggesting that this treatment dosage achieves sufficient brain levels to effectively and specifically engage the target in our model.

### Chronic FKBP51 inhibition modulates the HPA axis and slows hindlimb clasping deficit in PS19 mice in a sex-dependent manner

**Fig. 2:**
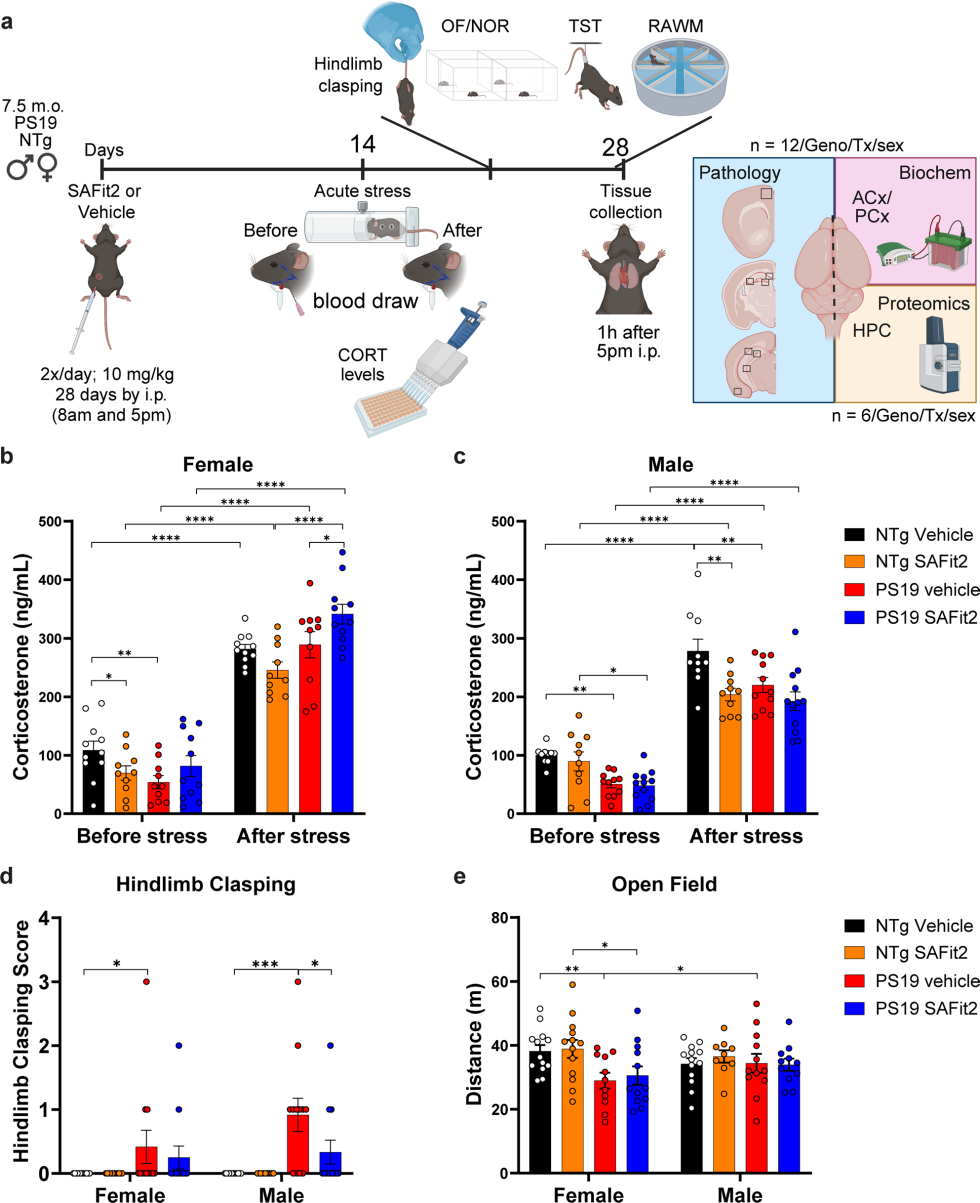
Chronic FKBP51 inhibition modulates HPA axis dynamics and slows hindlimb clasping deficit in a sex-dependent manner.** a** Schematic timeline of the experimental design. 7.5-month-old male and female non-transgenic (NTg) and PS19 mice received intraperitoneal (i.p.) injections of SAFit2 (10 mg/kg) or vehicle twice daily for 28 days. Acute restraint stress and blood collection were performed on day 14. Behavioral assays were conducted during the final week. Brains were collected 1 h after the final dose; the left hemisphere was processed for pathology and the right for biochemistry (anterior and posterior cortex, ACx/PCx) and proteomics (hippocampus, HPC). Serum corticosterone levels (ng/mL) measured by ELISA in (**b**) females and (**c**) males before (basal) and 30 min after 10 min tube restraint stress. **d** Hindlimb clasping scores assessing neurodegenerative progression. Severity was graded on a 0–3 scale, where 0 represents a normal reflex and 3 indicates severe postural abnormality. **e** Total distance (m) traveled in the Open Field (OF) test. Data are presented as mean ± SEM. *n* = 9–13 mice per sex/genotype/treatment (indicated by individual points within bars). Exact final sample sizes for each experimental group in (**d**, **e**) are detailed in Supplementary Table 15. Statistical significance was determined by three-way ANOVA (Sex x Genotype x Treatment) or Repeated Measures ANOVA (for serum CORT), followed by Bonferroni’s post-hoc test. Detailed statistics are provided in Supplementary Table 4. *p < 0.05, **p < 0.01, ***p < 0.001, ****p < 0.0001

Based on effectiveness of previous studies and our confirmation of brain penetrance, the therapeutic potential of FKBP51 inhibition in the context of tauopathy was evaluated. To this end, PS19 and NTg littermates were treated with SAFit2 (10 mg/kg) or vehicle twice daily for 4 weeks at 7.5-month-old (Fig. 2a), when tau pathology is already starting to accumulate in this model [51]. To verify *in* vivo systemic target engagement of SAFit2, the functionality of the hypothalamic-pituitary-adrenal (HPA) axis was assessed. Given the role of FKBP51 in glucocorticoid receptor (GR) regulation [52], we measured serum corticosterone (CORT) levels before and after a 10-min tube restraint stress. Including this readout allowed us to not only confirm drug activity, but also to evaluate whether potential behavioral recoveries in PS19 mice were dependent on canonical systemic stress hormone suppression or driven by independent mechanisms. In NTg mice, SAFit2 treatment induced distinct, sex-specific physiological responses. Basal morning CORT levels were significantly reduced in SAFit2 treated NTg females (*p* = 0.028, Fig. 2b), suggesting an optimization of the negative feedback loop. In contrast, NTg males exhibited a strongly attenuated CORT response only following acute stress (*p* = 0.001, Fig. 2c), consistent with increased stress resilience, as has been previously demonstrated in male wild-type mice treated with SAFit2 [24]. A distinct genotype-dependent effect was observed in the PS19 tauopathy model, where male PS19 mice showed no change, but PS19 females treated with SAFit2 exhibited significant increased post-stress CORT compared to vehicle controls (*p* = 0.025, Fig. 2b, c).

Next, gross neurological status was assessed using the hindlimb clasping test. Consistent with the progressive motor deficits previously described for this model [51], vehicle-treated PS19 mice exhibited significant hindlimb clasping compared to NTg controls (female *p* = 0.041 and male *p* = 0.0003, Fig. 2d). Notably, SAFit2 treatment significantly reduced the clasping score in PS19 males (*p* = 0.020, Fig. 2d), suggesting a rescue of the neurodegenerative phenotype. Subsequently, to ensure that performance in cognitive tasks and motor reflexes was not confounded by locomotor alterations or altered affective states, general activity, mean speed and time spent in center in the Open Field test were evaluated. While PS19 females showed a reduction in locomotion (vehicle *p* = 0.001 and SAFit2 *p* = 0.016) and mean speed (vehicle *p* = 0.007 and SAFit2 *p* = 0.015) compared to NTg controls, no significant effect of SAFit2 treatment was observed by sex or genotype in either distance traveled (Fig. 2e) or mean speed (Supplementary Fig. 1a). Similarly, time spent in the center was comparable across all groups (Supplementary Fig. 1b), ruling out baseline differences in anxiety-like behavior. Therefore, SAFit2 treatment specifically slows the hindlimb clasping phenotype in male PS19 mice, without altering general locomotor activity or basal anxiety. This confirms that the improved clasping score reflects a true rescue of motor neuron function rather than a non- specific behavioral confound.

### SAFit2 treatment slows cognitive deficits and depressive-like behavior in male PS19 mice

Next, recognition memory was evaluated using the Novel Object Recognition (NOR) task. To ensure the validity of the discrimination index, a strict inclusion criterion was applied: only mice with a total exploration time of > 10 s during the test phase were included in the analysis (Supplementary Fig. 1c, d). This quality control resulted in a final sample size of *n* = 3–9 per group for most cohorts.

**Fig. 3:**
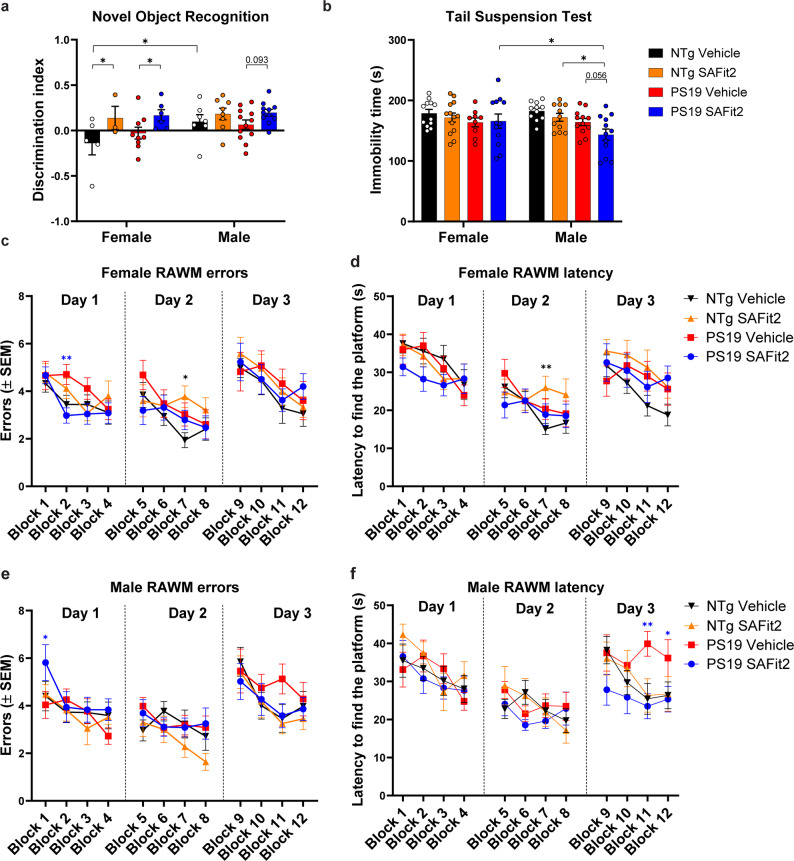
SAFit2 treatment preserves cognitive function and flexibility in PS19 mice. Behavior results from NTg and PS19 mice, comparing SAFit2 vs. vehicle treatment. **a** Discrimination index in the Novel Object Recognition (NOR) task. Scores > 0 indicate a preference for the novel object, while scores ≤ 0 indicate no discrimination. A strict total exploration time of at least 10 s was used as an exclusion criterion for this test, leaving an n of 3–9 for females and 7–12 for males (indicated by individual points). **b** Total immobility time in seconds (s) during the last 4 min of the 6-min Tail Suspension Test (TST) assessing depressive-like behavior. *n* = 9–13 mice per group (indicated by individual points). Radial Arm Water Maze (RAWM) was used to evaluate spatial working memory and cognitive flexibility. Days 1–2 represent the acquisition phase; Day 3 represents reversal learning (new platform location). Errors and latency to find the platform in seconds (s) in (**c**, **d**) females and (**e**, **f**) males across 3 days are shown. Exact final sample sizes for each experimental group are detailed in Supplementary Table 15. Data are presented as mean ± SEM. Statistical significance was determined by three-way ANOVA (Sex x Genotype x Treatment) for panels **a**, **b** and Repeated Measures ANOVA for **c**-**f**, followed by Bonferroni’s multiple comparisons test. Detailed statistics are provided in Supplementary Table 4. *p < 0.05, **p < 0.01

In the female cohort, vehicle-treated mice of both genotypes failed to discriminate between objects, exhibiting indices near or below zero (Fig. 3a). Remarkably, SAFit2 treatment significantly improved the discrimination index in both NTg (*p* = 0.046) and PS19 (*p* = 0.049) females compared to vehicle treated females (Fig. 3a). Although it did not reach significance, SAFit2 treatment in PS19 male mice showed a trending effect (*p* = 0.093) toward improving the discrimination index (Fig. 3a). To assess depressive-like behavior and stress coping, which has been shown to be improved in FKBP51 KOs and by SAFit2 treatment previously [53, 54], Tail Suspension Test (TST) was used. Importantly, the latency to first immobility did not differ across any of the groups (Supplementary Fig. 1e), confirming that the initial motivational drive to escape was preserved and unaffected by sex, genotype or treatment. While no differences in total immobility time were observed in females, PS19 males treated with SAFit2 presented a reduction in immobility time (Fig. 3b). Although this did not reach significance compared to PS19 vehicle controls (*p* = 0.056), SAFit2 treated PS19 males spent significantly less time immobile compared to their treated NTg counterparts (*p* = 0.010). This suggests that SAFit2 reduces behavioral despair specifically in the context of tauopathy in male mice, independent of baseline motivation.

Spatial working memory and cognitive flexibility were assessed using the Radial Arm Water Maze (RAWM). In the female cohort, a transient treatment effect in the NTg mice was observed. Specifically, NTg females treated with SAFit2 exhibited increased errors (*p* = 0.004, Fig. 3c) and latency (*p* = 0.005, Fig. 3d) during the consolidation phase on Day 2 compared to vehicle controls, while PS19 females did not show changes in the RAWM task regardless of treatment. In contrast, the male cohort revealed a robust therapeutic benefit. All male groups demonstrated effective learning during the acquisition phase (Days 1–2), with the SAFit2 treated NTg males showing the least number of errors (Fig. 3e) and latency (Fig. 3f) in the Day 2 testing phase. During the Reversal Learning phase on Day 3, a striking divergence emerged where the SAFit2 treated PS19 mice were able to find the relocated platform similarly to the NTg controls. As expected, vehicle treated PS19 males showed a significantly higher latency (*p* = 0.003) to find the platform along with a higher number of errors (*p* = 0.059) even in late blocks (Fig. 3e, f). These results suggest that FKBP51 inhibition preserves cognitive flexibility, specifically in PS19 males.

### SAFit2 promotes the clearance of multimeric AT8 tau in female PS19 mice

**Fig. 4:**
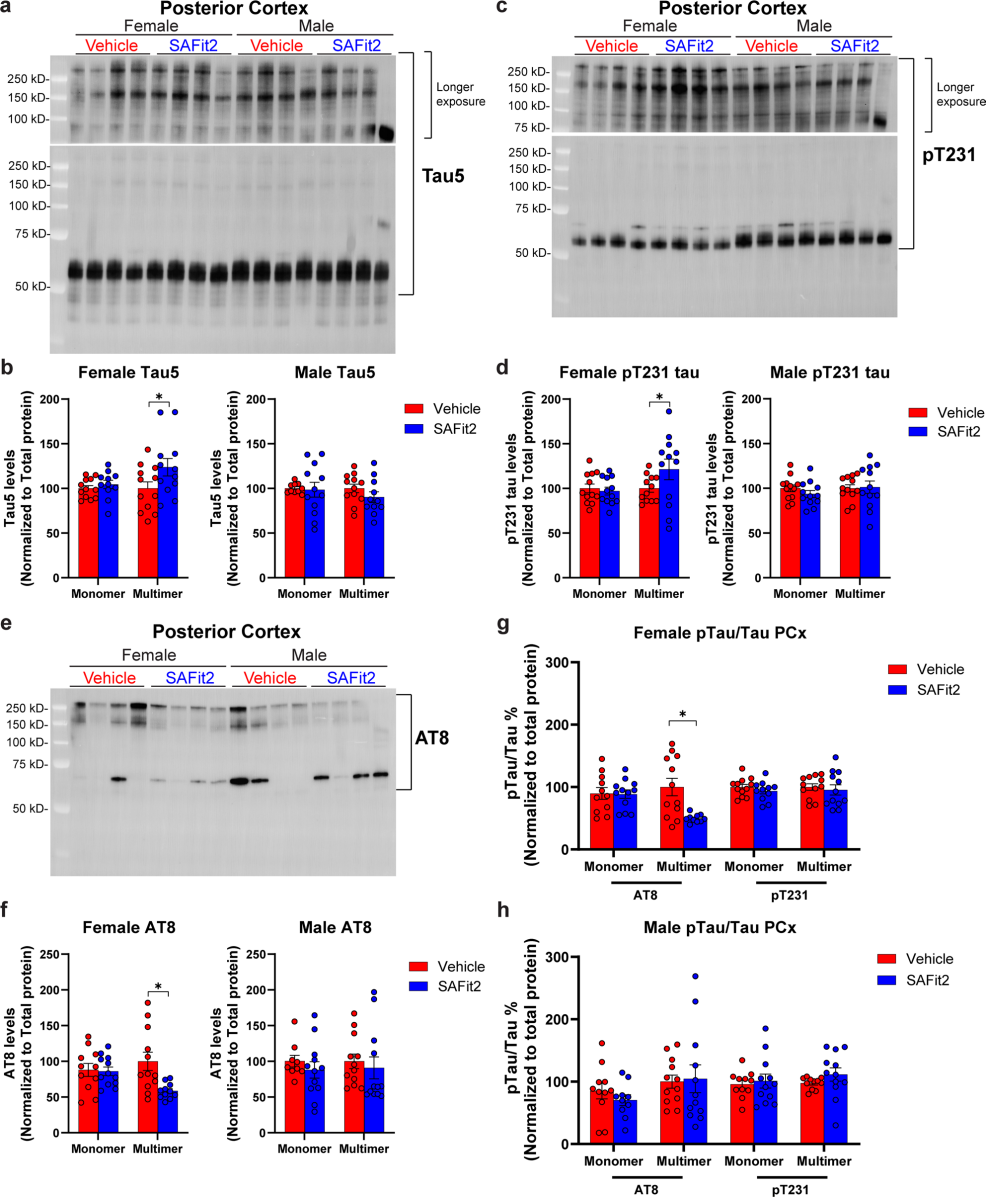
Sex-divergent response of SAFit2 on multimeric tau species in the Posterior Cortex (PCx). Biochemical analysis of monomeric (~ 68 kDa) and multimeric (≥ 75 kDa) tau species from semi-denaturing western blots with PCx homogenates from PS19 mice. Representative blots and quantification of (**a**, **b**) Total Tau (Tau5), (**c**, **d**) pT231 tau, and (**e**, **f**) AT8 tau (pS202/pT205) are shown. Ratios of pTau to Total Tau (AT8/Tau5 and pT231/Tau5) for the monomeric and the multimeric fraction from (**g**) females and (**h**) males. Protein levels were normalized to stain-free total protein displayed in Suppl. Figure 4. Data are presented as mean ± SEM. *n* = 11–12 mice per group. Statistical significance was determined by two-way ANOVA (Sex × Treatment) followed by Bonferroni’s multiple comparisons test. Non-normally distributed datasets were analyzed using the Kruskal-Wallis test followed by Dunn’s multiple comparisons test. *p < 0.05 vs. Vehicle. Detailed statistics are provided in Supplementary Table 6

To dissect the molecular impact of FKBP51 inhibition on tau dynamics, total tau and phosphorylated tau in the anterior cortex (ACx, Supplementary Fig. 2a-h, Supplementary Fig. 4a-c), posterior cortex (PCx, Fig. 4a-h, Supplementary Fig. 4d-f) and hippocampus (HPC, Supplementary Fig. 3a-h, Supplementary Fig. 4g-i) was evaluated from the treated PS19 mice using semi-denaturing western blots. This method supports the differentiation between monomeric (~ 68 kDa) and high-molecular-weight (HMW) tau multimers (≥ 75 kDa) [55, 56].

In the ACx, analysis of total tau by Tau5 revealed that monomeric and multimeric tau was not altered by SAFit2 treatment regardless of sex (Supplementary Fig. 2a, b). Similarly, probing for pT231 tau showed no significant treatment-dependent changes in either the monomeric or multimeric fractions (Supplementary Fig. 2c, d). However, reductions of the AT8-positive (pS202/pT205) tau multimers were measured specifically in female PS19 mice (*p* = 0.014) (Supplementary Fig. 2e, f). Also, unlike the Tau5 and pT231 tau antibodies, most of the signal for AT8 was found to be recognizing SDS-stable multimeric tau (≥ 75 kDa). To ensure epitope specificity independent of tau levels, the phospho-tau to total tau ratios for each antibody were analyzed. This confirmed a significant decrease in the multimeric AT8/Tau5 ratio in females (*p* = 0.028) (Supplementary Fig. 2g), whereas pT231 ratios remained unaltered regardless of treatment or sex in the ACx (Supplementary Fig. 2g, h).

Analysis of the PCx revealed a more complex, sex-divergent response. In males, SAFit2 treatment did not significantly alter the levels of monomeric or multimeric tau species compared to vehicle controls (Fig. 4a-h). Conversely, females exhibited a specific remodeling of the multimeric pool. SAFit2 treatment induced a significant increase in multimeric total tau levels (*p* = 0.024) (Fig. 4a, b). While absolute levels of multimeric pT231 tau also increased (*p* = 0.045) (Fig. 4c, d), multimeric AT8-positive tau levels were significantly reduced (*p* = 0.026) (Fig. 4e, f), which complements the reduction in the ACx. Critically, subsequent ratio analysis revealed that the multimeric pT231/Tau5 ratio remained unchanged indicating that the rise in pT231 merely reflected the total protein increase rather than a specific alteration in phosphorylation stoichiometry at this site. In contrast, the multimeric AT8/Tau5 ratio was significantly decreased in female PS19 mice (*p* = 0.042) (Fig. 4g). No significant changes in tau were observed in males (Fig. 4h).

Evaluation of the HPC revealed a distinct regional response to SAFit2 (Supplementary Fig. 3a-h). Total monomeric and multimeric tau (Tau5), as well as pT231 tau, were robustly preserved and remained unaltered by treatment in both sexes (Supplementary Fig. 3a-d). Interestingly, probing with the AT8 antibody revealed that while females displayed only a non-significant trend toward reduced multimeric levels (*p* = 0.086), SAFit2-treated males exhibited a significant reduction in AT8-positive multimers (Supplementary Fig. 3e, f). However, subsequent ratio analysis indicated no significant overall changes in phosphorylation stoichiometry for either AT8 or pT231 relative to total tau in this region (Supplementary Fig. 3g, h). These findings suggest that at this 8.5-month disease stage, the biochemical clearance of pathological tau is more pronounced in the cortex compared to the hippocampus.

Finally, to rigorously validate basal expression across conditions, PCx homogenates were also evaluated under fully denaturing and reducing conditions (boiled at 100 °C with β-mercaptoethanol) to focus exclusively on the monomeric tau pool (Supplementary Fig. 5a-f). Consistent with our semi-denaturing observations, female PS19 mice showed no changes in total monomeric tau (Tau5) or its phosphorylation state at AT8 or pT231 sites (Supplementary Fig. 5b-f). Interestingly, a slight but significant reduction in total monomeric tau levels was measured in male SAFit2-treated mice (Supplementary Fig. 5b, p = 0.042). Since absolute levels of monomeric phosphorylated AT8 and pT231 also remained unchanged (Supplementary Fig. 5c, e) for male mice, this lower total tau protein level resulted in a significant increase in the pT231/Tau5 ratio (Supplementary Fig. 5f, p = 0.044). Taken together, these data demonstrate that while basal monomeric tau dynamics can show subtle, sex-divergent shifts, the therapeutic effect of SAFit2 in female PS19 mice is strictly driven by the selective clearance of AT8-positive tau multimers, without fundamentally altering physiological monomeric tau expression.

### SAFit2 treatment significantly reduces TOC1-positive tau oligomers in male PS19 mice

To investigate the spatial distribution of tau pathology, immunofluorescence analysis across key brain regions involved in memory processing was performed, including the prefrontal cortex (Cx), dorsal (d) and ventral (v) hippocampus (CA1, CA3, DG), and entorhinal cortex (ECx). In female PS19 mice, SAFit2 treatment resulted in a localized significant reduction in total tau levels in the vCA3 region (*p* = 0.044), while total tau in other hippocampal or cortical areas remained unchanged (Supplementary Fig. 6a, b). In contrast, SAFit2 treatment in PS19 male mice showed no change in total tau levels across all regions examined (Supplementary Fig. 6c, d), consistent with biochemical analysis.

SAFit2 treatment did not alter pT231 tau in PS19 females (Supplementary Fig. 7a, b). Interestingly, treated PS19 males exhibited a significant increase in pT231 tau in the dCA3 (*p* = 0.025) and a strong trend towards an increase in the vCA1 (*p* = 0.051) and vDG (*P* = 0.065, Supplementary Fig. 7c, d), suggesting a focal accumulation of this intermediate tau species. Notably, qualitative assessment of the pT231 staining revealed a distinct distribution pattern beyond the neuronal compartment. Across all experimental groups and brain regions, a widespread pT231 immunoreactivity exhibiting an astrocytic-like morphology was observed. To confirm the specificity of the antibody, complementary staining was performed in tissues from Tau KO and hTau mice (Supplementary Fig. 8a, b), which showed minimal reactivity or expected distribution, respectively. The glial-like pT231 tau staining in this study are distinct from the somatic staining typical of neurons and appeared independent of sex or treatment, indicating that accumulation of pT231 tau in the glia may be a consequence of the vehicle used to solubilize SAFit2.

**Fig. 5:**
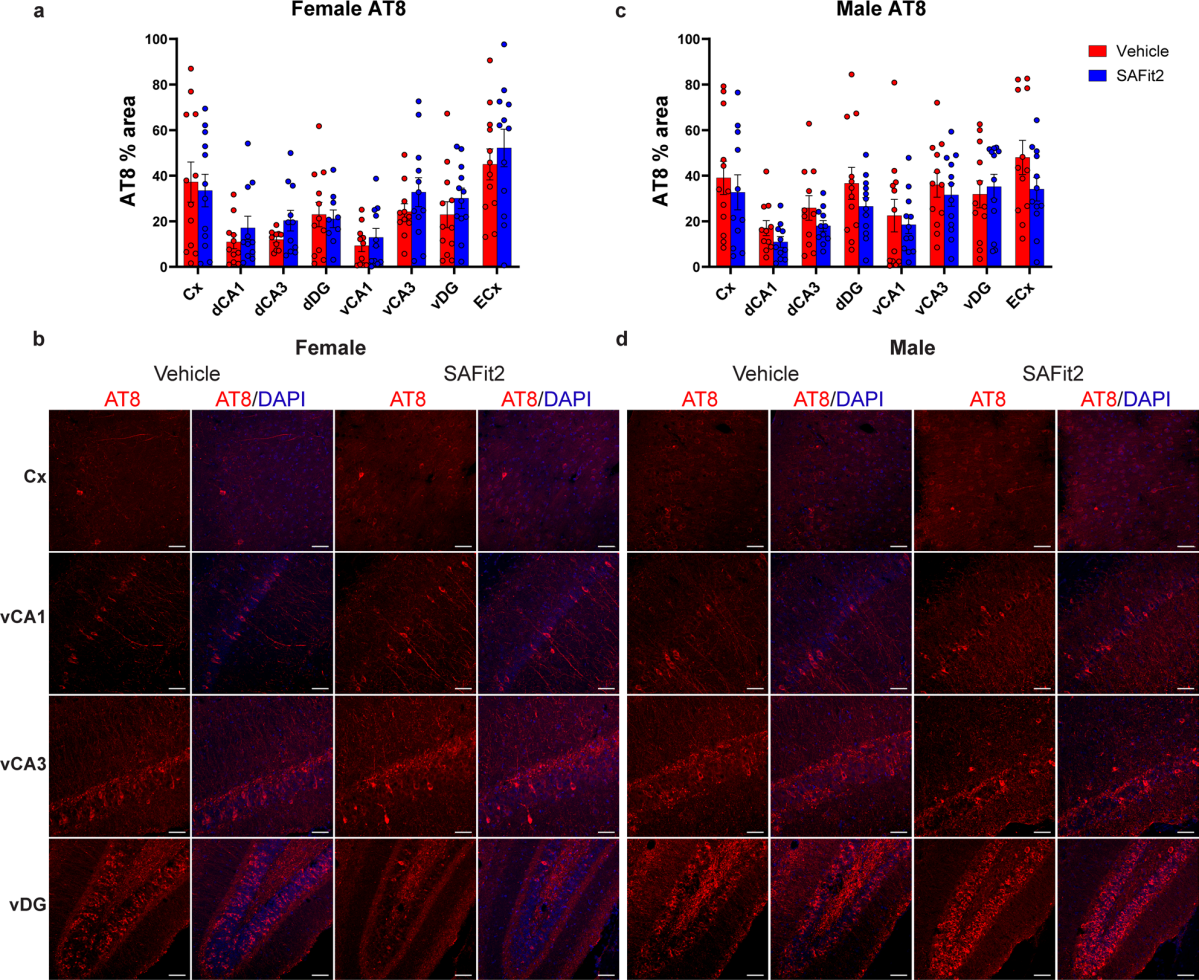
SAFit2 treatment does not alter AT8-positive tau aggregates in PS19 mice. Quantification of the percentage area and select representative images of AT8 immunoreactivity (red) for (**a**, **b**) female and (**c**, **d**) male PS19 mice following 28 days of SAFit2 treatment. Quantitative analysis across dorsal and ventral hippocampal subfields (dCA1, dCA3, dDG, vCA1, vCA3, vDG), and cortical regions (Cx, ECx) of SAFit2-treated (blue bars) compared to vehicle-treated (red bars) groups. Representative images for other regions can be visualized in Supplementary Fig. 9a. Data are presented as mean ± SEM with individual data points (*n* = 10–12 mice/group). Statistical significance was determined by two-way ANOVA (Sex × Treatment) followed by Bonferroni’s multiple comparisons test. Non-normally distributed datasets were analyzed using the Kruskal-Wallis test followed by Dunn’s multiple comparisons test. DAPI nuclei stain is shown in blue, scale bars: 50 μm. Detailed statistics are provided in Supplementary Table 10

**Fig. 6:**
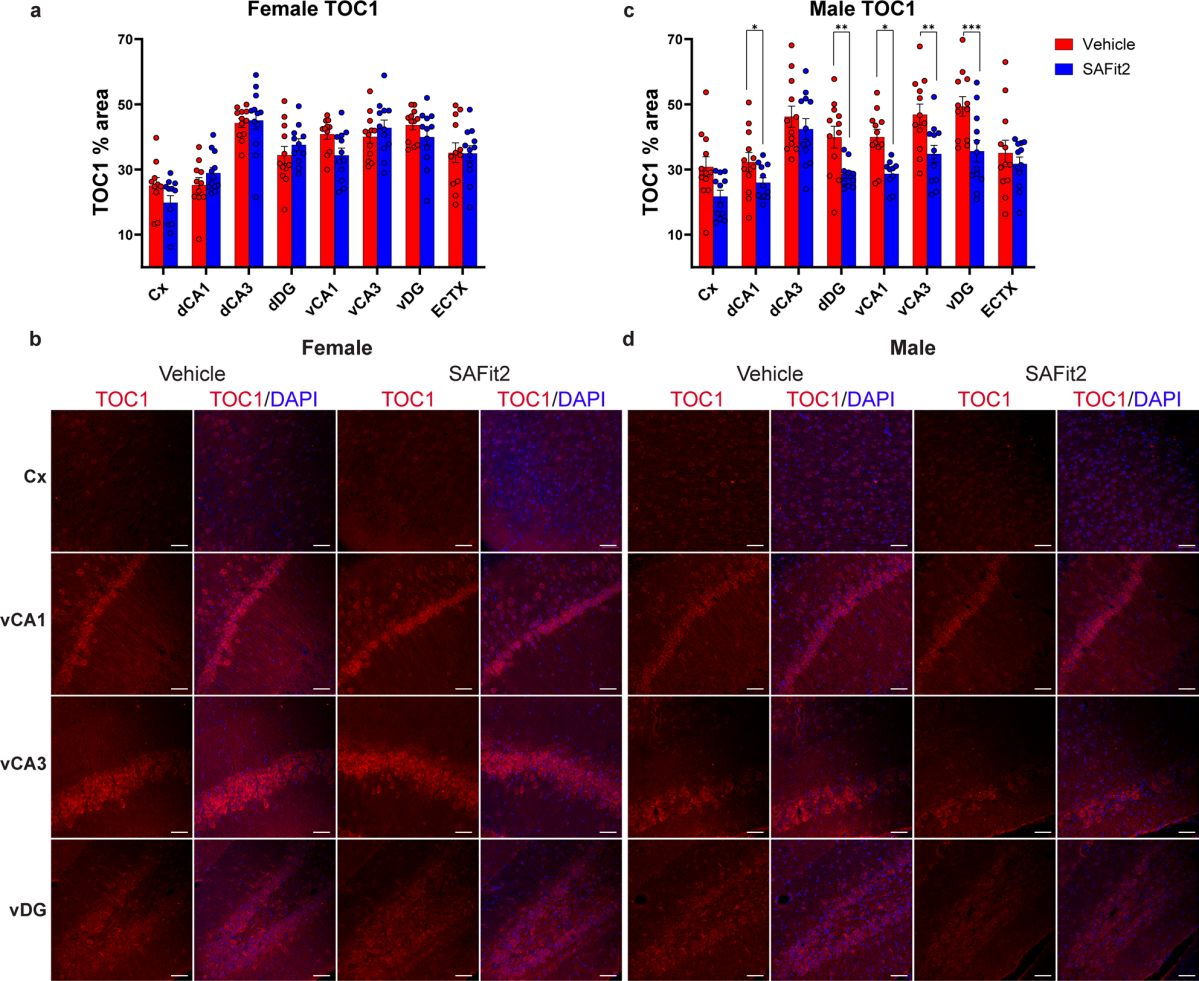
Sex-specific clearance of TOC1-positive tau oligomers following FKBP51 inhibition. Quantification of the percentage area and select representative images of TOC1 immunoreactivity (red) for (**a**, **b**) female and (**c**, **d**) male PS19 mice SAFit2-treated (blue bars) compared to vehicle controls (red bars). Analysis of TOC1-positive species was performed across prefrontal cortex (Cx), dorsal and ventral hippocampal subfields (dCA1, dCA3, dDG, vCA1, vCA3, vDG), and entorhinal cortex (ECx). Representative images for other regions can be visualized in Supplementary Fig. 9b. Data are presented as mean ± SEM with individual data points (*n* = 10–12 mice/group). Statistical significance was determined by two-way ANOVA (Sex × Treatment) followed by Bonferroni’s multiple comparisons test. Non-normally distributed datasets were analyzed using the Kruskal-Wallis test followed by Dunn’s multiple comparisons test. *p < 0.05, **p < 0.01, ***p < 0.001. DAPI nuclei stain is shown in blue, scale bars: 50 μm. Detailed statistics are provided in Supplementary Table 11

SAFit2 treatment did not alter the levels of AT8-positive tau (Fig. 5a-d, Supplementary Fig. 9a), which is widely used to assess pathological tau in postmortem brain tissue [57]. This divergence from our biochemical findings likely reflects the distinct tau pools interrogated by each assay, with immunofluorescence predominantly capturing thermodynamically stable fibrillar deposits rather than soluble intermediate multimers. However, probing for TOC1, an antibody specific for oligomeric tau species, revealed a striking therapeutic effect in a sex-dependent way. While TOC1 levels remained unchanged in female PS19 mice (Fig. 6a, b), SAFit2 treatment significantly reduced TOC1-positive tau oligomers in male PS19 mice across multiple regions, including the dorsal (CA1 and DG) and ventral hippocampus (CA1, CA3, and DG) (Fig. 6c, d, Supplementary Fig. 9b). Collectively, these histological findings complement the biochemical data, suggesting that SAFit2 primarily targets oligomeric and intermediate tau species and this effect is dependent on sex.

### SAFit2 treatment enhances markers of astrocytes in the ventral hippocampus

Given the close relationship between tau pathology and neuroinflammation [51], markers of astrocytes (GFAP) and microglia (Iba-1) were evaluated. As expected, a robust genotype effect was observed across both sexes, where PS19 mice exhibited widespread and significant increases in GFAP (Supplementary Fig. 10a-d) and Iba-1 (Supplementary Fig. 11a-d) immunoreactivity compared to NTg controls in the hippocampus and cortex. Overall, in female PS19 mice treated with SAFit2, there was lower GFAP signal that neared significance for the vDG (*p* = 0.055) (Supplementary Fig. 10a, b). Surprisingly, SAFit2 treatment in male PS19 mice significantly enhanced GFAP staining in the vDG (*p* = 0.007) (Supplementary Fig. 10c, d), while other hippocampal subregions and the cortex were not affected. Iba-1 immunoreactivity was not altered by SAFit2 treatment in PS19 mice regardless of sex (Supplementary Fig. 11a-d). Interestingly, a significant reduction in Iba-1 levels in the cortex of SAFit2 treated NTg females was observed (*p* = 0.043) (Supplementary Fig. 11a, b), but this reduction was limited to a single region.

### SAFit2 modulates FKBP51 levels in a sex-dependent manner

**Fig. 7:**
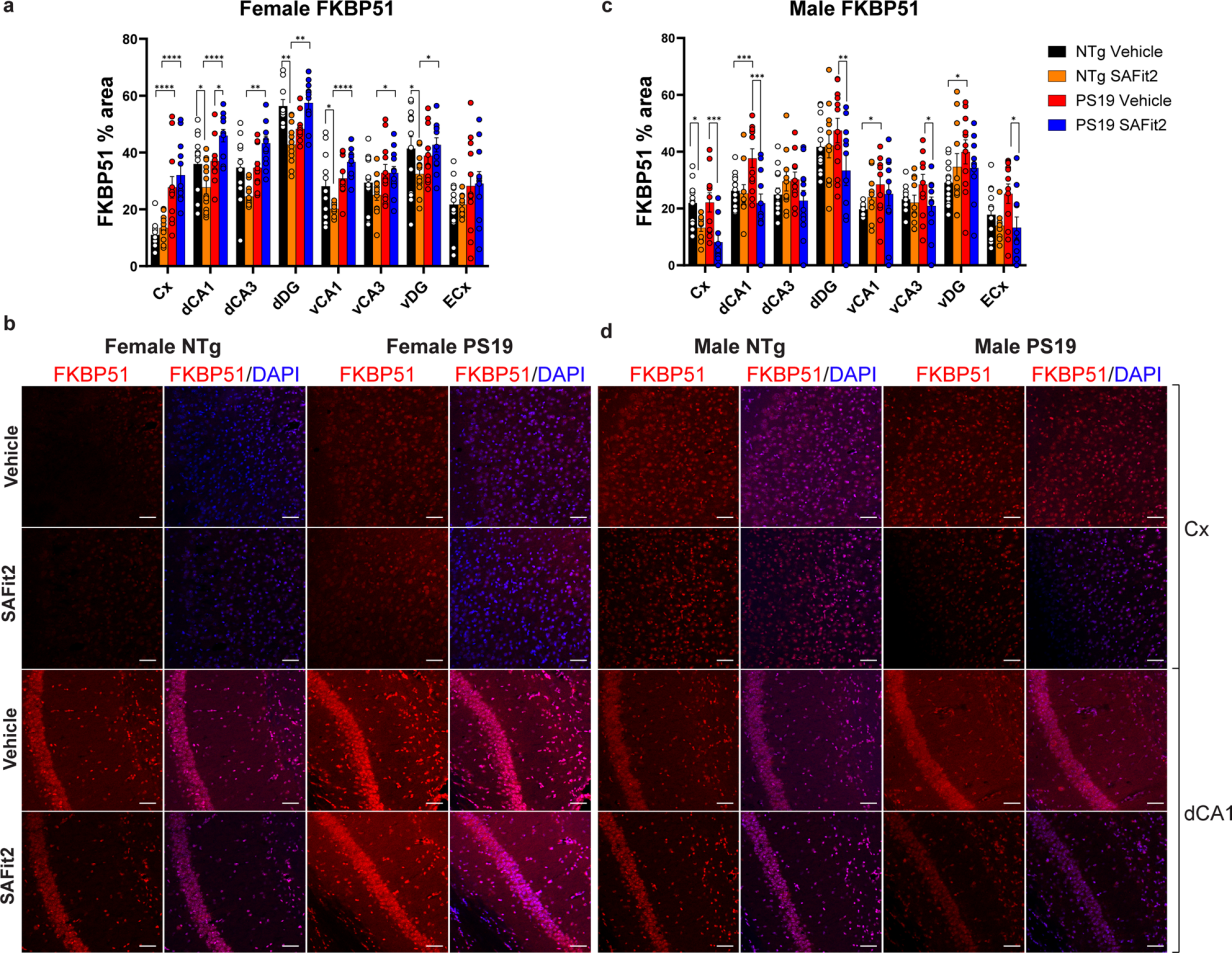
Sex-divergent regulation of FKBP51 protein levels following chronic pharmacological inhibition. Quantitative analysis and select representative images of FKBP51 immunoreactivity (red) in the hippocampus and cortex of (**a**, **b**) female and (**c**, **d**) male NTg and PS19 mice following 28 days of SAFit2 treatment. Analysis was performed in prefrontal Cortex (Cx), dorsal and ventral hippocampal subfields (dCA1, dCA3, dDG, vCA1, vCA3, vDG), and Enthorinal cortex (ECx), comparing SAFit2 treatment (orange and blue bars) with vehicle-treated controls (black and red bars). Representative images for other regions can be visualized in Supplementary Fig. 12. Data are presented as mean ± SEM with individual data points (*n* = 10–12 mice/group). Statistical significance was determined by three-way ANOVA (Sex × Treatment × Genotype) followed by Bonferroni’s multiple comparisons test. Non-normally distributed datasets were analyzed using the Kruskal-Wallis test followed by Dunn’s multiple comparisons test. *p < 0.05, **p < 0.01, ***p < 0.001, ****p < 0.0001. DAPI nuclei stain is shown in blue, scale bars: 50 μm. Detailed statistics are provided in Supplemental Table 14

To characterize the local target landscape and determine whether the PS19 genotype or chronic SAFit2 treatment alters target availability, FKBP51 immunoreactivity across the hippocampus and cortex was quantified (Fig. 7a-d, Supplementary Fig. 12). Comparisons between vehicle treated groups revealed region-specific increases by genotype in FKBP51 in female (Cx, *p* < 0.0001) and male (vCA1, *p* = 0.022 and vDG, *p* = 0.023) PS19 mice compared to Ntg littermates. Evaluation of SAFit2 treatment revealed distinct responses between sexes. A significant reduction was observed in the hippocampus (dCA1, *p* = 0.032; dDG, *p* = 0.005; vCA1, *p* = 0.031 and, vDG, *p* = 0.049) of NTg females, suggesting that SAFit2 can modulate basal FKBP51 levels. However, the PS19 females were largely resistant to this regulation, with most regions showing no changes and a significant increase that was restricted to the dCA1 region (*p* = 0.029) (Fig. 7a, b). In contrast, male PS19 mice were responsive to treatment, where SAFit2 induced a significant downregulation of FKBP51 in the Cx (*p* = 0.0004), hippocampus (dCA1 *p* < 0.0001, dDG *p* = 0.003, vCA3 *p* = 0.048), and ECx (*p* = 0.012) (Fig. 7c, d); while male NTg presented a decrease only in the Cx (*p* = 0.019). These data suggest that chronic SAFit2 treatment can alter the levels of FKBP51 in a sex and genotype dependent manner.

### Hippocampal proteomics reveals distinct molecular signatures underlying the sex-specific response to SAFit2

**Fig. 8:**
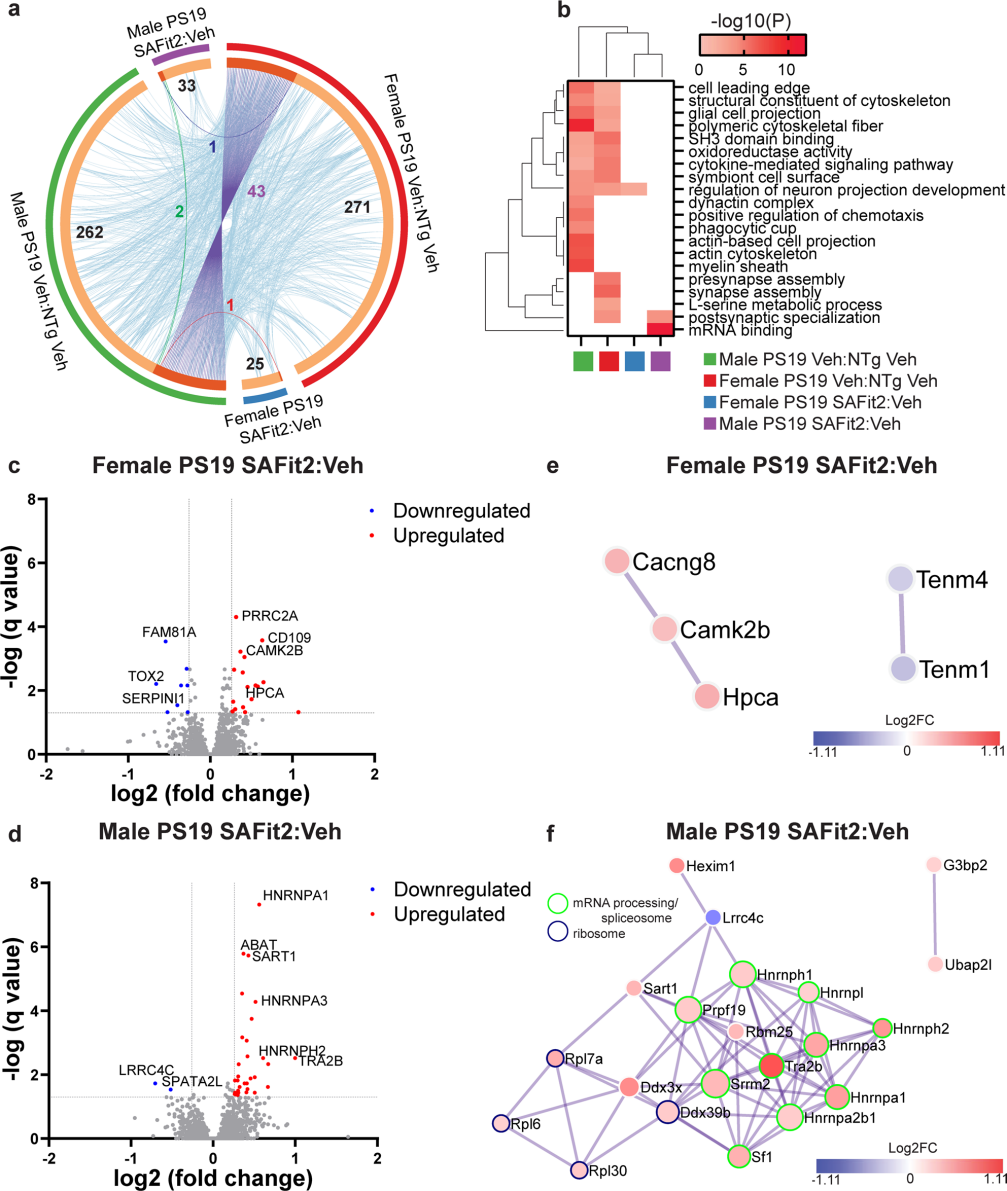
Sex-specific profile of the hippocampal proteome following SAFit2 treatment.** a** Circos plot illustrating the landscape of differentially expressed proteins (DEPs) across four key comparisons: the effect of genotype (PS19 Vehicle vs. NTg Vehicle) and the effect of treatment (PS19 SAFit2 vs. PS19 Vehicle and NTg SAFit2 vs. NTg Vehicle) in males and females, except in male NTg mice, since no DEPs were identified. The outer ring represents the total number of unique and shared DEPs for each group, and the inner ring highlights the identical proteins (dark orange) connected by colored lines. Light blue lines represent shared Gene Ontology (GO) terms. **b** Heatmap of hierarchically clustered GO terms (GO Biological Process, GO Molecular Function, and GO Cellular Component) enriched for the same comparisons in panel a. Color intensity represents statistical significance (-log10 P-value) with white denoting absence of enrichment. Volcano plots showing the differential expression analysis of the hippocampal proteome in SAFit2-treated PS19 (**c**) females and (**d**) males relative to vehicle controls. The x-axis represents Log2 Fold change (log2FC), and the y-axis represents significance (-log(q-value)). Red dots indicate significantly upregulated proteins (log2FC > 0.26); blue dots indicate downregulated proteins (log2FC < -0.26). Key hits are labeled. Protein-protein interaction (PPI) networks of significant hits in SAFit2 treated (**e**) female and (**f**) male PS19 mice. No MCODE networks were identified in females. In males, MCODE networks identified enrichments in mRNA processing and splicing (green halo), and ribosomal function (dark blue halo) are shown. Node color indicates the magnitude of upregulation (red) or downregulation (blue)

Given the striking sex divergence observed in behavioral, biochemical, and histological outcomes, the global molecular landscape driving these differences was characterized by 4D proteomic analysis of hippocampal tissue from NTg and PS19 mice treated with vehicle or SAFit2. Of the over 10,000 proteins identified, differentially expressed proteins (DEPs) were identified using a significance threshold of q < 0.05 and a fold change of > 1.2 (upregulated) or < -1.2 (downregulated). The complexity of the proteomic remodeling is illustrated in the Circos plot (Fig. 8a). The PS19 genotype drove the most extensive changes, with a comparable magnitude of disease-associated alterations in both sexes: 262 DEPs in male PS19 mice and 271 DEPs in female PS19 mice. While there are many shared ontology terms between these changed proteins, as indicated by the light blue lines, 43 DEPs were identical between the male and female PS19 mice compared to their respective NTg littermates.

In response to SAFit2, the modulation was more targeted and subtle compared to the extensive genotype effect. SAFit2 treatment in PS19 mice resulted in 33 DEPs in males and 25 DEPs in females compared to their vehicle treated counterparts (Fig. 8a). There was no overlap between the treatment-responsive proteins in PS19 males versus females, providing further evidence that SAFit2 engages distinct molecular mechanisms in each sex. Female NTg treated with SAFit2 had 37 DEPs, none of which overlapped with SAFit2 treated PS19 females, but 2 overlapped with SAFit2 treated PS19 males (Fig. 8a). Given these limited overlaps, it appears that the proteomic changes observed in PS19 mice are unique to the combination of FKBP51 inhibition and the tauopathic setting.

To decipher the biological impact of these changes, Gene Ontology (GO) enrichment analysis, focusing on biological processes and molecular functions was performed. The heatmap reveals that the most profound functional alterations are driven by the presence of pathological tau (genotype effect; green and red columns, Fig. 8b). Both male (green) and female (red) vehicle treated PS19 mice exhibited strong enrichment for terms related to neuronal integrity, such as ‘structural constituent of cytoskeleton’ and ‘polymeric cytoskeletal fiber’. This confirms a shared alteration of the cytoskeleton architecture. However, some distinct sex-specific patterns also emerged with vehicle treated PS19 males showing specific enrichment in pathways related to myelin sheath, while females were characterized by alterations in pathways related to synaptic processes. In contrast, the SAFit2 treatment groups (blue and purple columns) show minimal enrichment terms. Male SAFit2 treated PS19 mice show alterations in mRNA binding and postsynaptic specialization (Fig. 8b). In contrast, female SAFit2 treated PS19 mice show enrichment of proteins related to the regulation of neuron projection development compared to their vehicle treated littermates (Fig. 8b). A closer look at the top 10 GO terms for the SAFit2 treated mice also highlights enrichment of stress granule assembly and cytosolic ribosome in SAFit2 treated male PS19 (Supplementary Fig. 13). The enrichment terms for SAFit2 treated PS19 females also expand to include regulation of synaptic plasticity and transporter activity pathways (Supplementary Fig. 13). In NTg females, the proteomic signature induced by SAFit2 treatment, covers activation of immune response, cytoskeletal and transport pathways, overlapping only with the regulation of synaptic plasticity and neuron to neuron synapse pathways observed the PS19 genotype (Supplementary Fig. 13). Overall, this suggests that SAFit2 modulates specific downstream pathways or distinct mechanisms by sex in PS19 mice.

To elucidate the specific molecular targets involved in these pathways, volcano plots for PS19 SAFit2-treated groups relative to vehicle controls were generated (Fig. 8c, d). Overall, the majority of SAFit2 treatment effects led to an upregulation of proteins, with a smaller subset of proteins being downregulated. Network analysis of the DEPs in SAFit2 treated PS19 females identified very few proteins that are known to physically interact (Fig. 8e). Multiple proteins related to synaptic plasticity were upregulated. These proteins included CaMKIIβ (CAMK2B), a central molecule in synaptic plasticity and memory formation [58], Hippocalcin (HPCA), a neuronal calcium sensor, and Calcium Voltage-Gated Channel Auxiliary Subunit Gamma 8 (CACNG8), a glutamate receptor regulatory protein. Concurrently, downregulation of trans-synaptic adhesion molecules, such as the Teneurins (TENM1, TENM4) was also observed. In contrast, the network analysis for SAFit2-treated PS19 males identified a robust and highly interconnected PPI module centered on mRNA processing/spliceosome and ribosome function (Fig. 8f). Among the top upregulated hits are key modulators of alternative splicing and mRNA processing, including several heterogeneous nuclear ribonucleoproteins (HNRNPA1, HNRNPA3, HNRNPH2), the Spliceosome Associated Factor 1 (SART1), and the splicing regulator, Transformer 2 Beta Homolog (TRA2B).

## Discussion

The immunophilin FKBP51 has emerged as a pivotal modulator of tau pathology [12, 59], yet its therapeutic validation in chronic, aged neurodegenerative models remains limited. In this study, the pharmacological inhibition of FKBP51 using a selective inhibitor, SAFit2, slowed cognitive deficits and depressive-like behavior in the PS19 mouse model of tauopathy. Crucially, our proteomic analysis uncovers a striking sexual dimorphism in the molecular mechanisms underlying this neuroprotection. While the therapeutic benefit in males is associated with a distinct modulation of RNA processing pathways and the significant clearance of TOC1-positive tau oligomers, females exhibit a preferential stabilization of synaptic networks, specifically restoring key drivers of plasticity such as Camk2b and Hpca. Thus, the divergent molecular pathways altered in PS19 mice by FKBP51 inhibition led to different functional and pathological outcomes by sex. This provides the first in vivo evidence that FKBP51 inhibition impacts the brain proteome through sex-specific remodeling.

Consistent with target engagement, chronic SAFit2 treatment improved behavioral deficits characteristic of the PS19 mouse model. Cognitive decline, particularly in spatial working memory and executive function, is a hallmark of tauopathy driven by hippocampal and cortical neurodegeneration [51, 60]. While SAFit2 demonstrated broad therapeutic benefits in male PS19 mice, including the slowing of motor deficits and preservation of spatial memory, its efficacy in females was highly task specific. As noted, SAFit2 failed to ameliorate motor pathology and spatial working memory in females, yet it significantly preserved recognition memory. This behavioral opposition aligns with our biochemical observations in females, where the clearance of pathological tau was most prominent in the cortex, a region critical for recognition memory, but less robust in the hippocampus. The potential mechanisms driving these regional and sex-specific biochemical differences are explored in greater detail below. This indicates that while widespread motor and spatial deficits may not be reversed at this advanced disease stage in females PS19, specific cognitive domains remain responsive to FKBP51 inhibition. Beyond cognition, neuropsychiatric symptoms, such as anxiety and depression, are common comorbidities in AD, which are exacerbated by tau pathology [61, 62]. SAFit2 treated PS19 male mice exhibited decreased behavioral despair in the TST without affecting the response to CORT. Crucially, our behavioral profiling confirmed that this rescue occurred without altering general locomotor activity, initial motivational drive, or basal anxiety. Similarly, female PS19 mice showed cognitive improvement despite elevated CORT levels. This dissociation strongly suggests that in the chronic neurodegenerative brain, the therapeutic benefit of SAFit2 is not primarily driven by a systemic suppression of the HPA axis, as might be predicted from canonical FKBP51 biology. Instead, the rescue appears to be mediated by central neuroprotective mechanisms, such as the stabilization of synaptic proteomes and the clearance of oligomeric tau species, which can occur independently of systemic glucocorticoid levels.

Dissecting the impact of SAFit2 on tau pathology revealed a notable dissociation between biochemical and immunohistochemical readouts. This highlights the complexity of targeting tau after pathogenic accumulation has begun. While western blot analysis demonstrated a significant reduction of AT8-positive multimers throughout the female PS19 cortex, immunofluorescence analysis of the same epitope showed no significant change. This discrepancy likely reflects the nature of the pathology at this stage of disease. In 8-month-old PS19 mice, AT8 immunoreactivity is largely driven by the accumulation of mature neurofibrillary tangles [51], dense aggregates that are thermodynamically stable and can persist even after the suppression of toxic soluble species [63]. In contrast, western blotting allows for the resolution of soluble multimers. Our data suggest that FKBP51 inhibition primarily targets these dynamic, intermediate species rather than disaggregating established fibrillar deposits. This interpretation is further supported by the male dataset, where reductions of TOC1-positive oligomers were measured. TOC1 recognizes tau dimers and larger oligomers [64], which have been linked to neurotoxicity [65–67]. Furthermore, our denaturing biochemical analyses confirmed that this targeted clearance occurs without broadly depleting the physiological monomeric tau pool. While a subtle reduction in total monomeric tau and a consequent shift in pT231 stoichiometry were noted specifically in treated males, the overall preservation of basal tau across both sexes emphasizes the conformational selectivity of SAFit2. This outcome is highly complementary to prior work from our lab demonstrating that overexpression of FKBP51 in the brains of another tau mouse model, rTg4510, promotes tau oligomerization, but does not change the levels of silver-positive, insoluble tau [12].

Collectively, these findings reveal a unified therapeutic mechanism for SAFit2: the targeted clearance of intermediate, neurotoxic tau aggregates. The contrast between the persistence of thermodynamically stable AT8-positive fibrillar pathology observed through staining and the selective reduction of soluble/intermediate tau species, AT8-positive multimeric species in females and TOC1-postivie tau oligomers in males, provides additional evidence towards these dynamic intermediate assemblies being more closely associated with motor and cognitive dysfunction than fibrillar pathology. This data adds to a growing list of papers demonstrating that intermediate tau species are more toxic than large tau aggregates [67–71]. By mitigating these specific soluble tau pools, SAFit2 facilitates distinct functional rescues even in the presence of established fibrillar burden. The specific pathological signature most susceptible to clearance varied by sex, In males, behavioral rescue coincided with reduced TOC1-positive oligomers, whereas in females SAFit2 more prominently reduced AT8-positive multimeric tau without broad rescue of motor or spatial memory deficits. This divergence may be due to: (i) the accelerated tauopathy characteristic of male PS19 mice [72], which may determine which tau conformers are most available for clearance at the time of intervention; (ii) differential regulation of FKBP51 by SAFit2, particularly the broader reduction of FKBP51 observed in male PS19 mice, which may favor destabilization of oligomeric tau species; and/or (iii) distinct downstream proteomic remodeling, with males showing enrichment of RNA processing/ribosomal pathways and females showing restoration of synaptic and calcium-signaling pathways. Together, these data support a model in which FKBP51 inhibition promotes clearance of the predominant dynamic pathogenic tau pool present in each sex, while stable fibrillar aggregates remain comparatively resistant over the treatment window used here. However, it is still unknown if the sex specific effects identified in this study are specific to SAFit2 or if other molecules that target FKBP51, like LA1011 [73], would have the same sexual dimorphism. The regional differences observed in total and pT231 tau following SAFit2 treatment may suggest that FKBP51-mediated clearance could be dependent on local chaperone levels and specific phosphorylation status of the tau species present.

By confirming HPA axis modulation and regional FKBP51 availability, this study suggests that the therapeutic responses in PS19 mice stem from disease-related dysregulation rather than a lack of drug activity. Indeed, evaluating this target landscape revealed genotype and sex dependent effects on FKBP51 regulation. While the mechanism of action for SAFit2 is to inhibit the PPIase activity of FKBP51 and not to alter the levels, in PS19 males and NTg mice, SAFit2 treatment resulted in a significant decrease of FKBP51 protein levels across cortical and hippocampal subfields. Therefore, SAFit2 may exert effects through both acute functional inhibition and secondary remodeling of FKBP51 abundance. We hypothesize that this unexpected reduction may occur through two distinct mechanisms. First, chronic ligand binding may induce conformational changes that destabilize the chaperone complex, becoming FKBP51 more susceptible to proteasomal degradation. Secondly, because FKBP51 is a key regulator of the glucocorticoid receptor, its sustained functional inhibition may trigger a compensatory downregulation of its own expression via feedback loops. FKBP51 levels are not often reported in SAFit2 treated mice, perhaps because of the difficulty in detecting endogenous FKBP51 in young mice, as most SAFit2 studies have used, so it is unclear if the reduction in FKBP51 levels following SAFit2 treatment is shared in other studies. It is important to note that male PS19 mice showed a greater decrease in FKBP51 levels by SAFit2 treatment than male NTgs. The widespread reduction of FKBP51 in male PS19 mice occurred concomitant with the clearance of TOC1 + oligomers. This suggests a direct link between lowering the cochaperone and reducing oligomeric tau stability. Given that FKBP51 has been demonstrated to colocalize with tau pathology [12], it is plausible that inhibiting FKBP51 facilitates the degradation of tau oligomers, which in turn may lead to the subsequent breakdown of FKBP51 associated with tau. It is not surprising that the greatest effect on tau was identified in the group with reduced FKBP51 levels. Prior work has demonstrated that ablation of FKBP51 enzymatic activity, the target of SAFit2, has little effect on tau stabilization compared to regulation of FKBP51 levels [59, 74].

Benefits of SAFit2 have been observed in other protein-misfolding models. In mouse models of HD and PD pathology, SAFit2 treatment has been shown to promote the clearance of toxic aggregates, presumably by re-engaging autophagy or proteasomal pathways suppressed by the chaperone complex [34, 75]. The clearance of TOC1 oligomers in our male cohort parallels these outcomes, reinforcing the concept that FKBP51 acts as a cross-disease regulator of amyloid stability.

While a prior study demonstrated that SAFit2 treatment reduces GFAP and IBA1 in the spinal cord of mice [28], the only significant change identified in the brains of SAFit2 treated NTg mice was in the cortex of females. This may suggest that SAFit2 treatment has a greater effect on inflammatory markers in the spinal cord than in the brain. Interestingly, the tau reductions by SAFit2 treatment did not resolve the robust neuroinflammation characteristic of the PS19 genotype. SAFit2 treatment in PS19 females slightly reduced markers of astrocytes in the vDG, but significantly increased GFAP in the same region in treated PS19 males. This uncoupling of neuroinflammatory markers and cognitive recovery is a surprising, but critical finding. This suggests that in the PS19 model, behavioral rescue can occur independently of a global reversal of gliosis. It further reinforces our conclusion that the primary driver of functional recovery is the reduction of tau oligomers rather than a secondary inhibition of the immune response.

Unbiased proteomic profiling in the hippocampus revealed that FKBP51 inhibition reversed the downregulation of critical synaptic drivers in the hippocampus. In females, this effect was characterized by the specific restoration of proteins essential for calcium signaling and long-term potentiation, CaMKIIβ and HPCA, reviewed in [76, 77]. This provides a molecular basis for the cognitive rescue observed in the NOR test, indicating that SAFit2 in females helps maintain synaptic plasticity pathways despite the presence of total tau burden.

Interestingly, the male proteome revealed a distinct but convergent pathway: the upregulation of RNA processing and ribosomal machinery. Specifically, a significant increase in heterogeneous nuclear ribonucleoproteins (hnRNPs), including Hnrnpa3, Hnrnph1 and Hnrnpa2b1, and the splicing regulator, TRA2B, was observed. This finding is particularly striking given that pathological tau species are known to sequester RNA-binding proteins, leading to their functional depletion and widespread splicing dysregulation [78]. More specifically, it has been reported that transcripts from these proteins are downregulated in PS19 mice compared to NTg [78] or are less associated with tau in the rTg4510 tau model [79]. However, another study shows a different finding that the Hnrnp2ab1 protein complexes with oligomeric tau in the brains of 3- and 6-month PS19 mice and that knocking down Hnrnpa2b1 can reduce the response to pathological tau in 4-month PS19 mice [80]. Despite the contrasting observation, the SAFit2-mediated upregulation of some hnRNP family members in our study may represent a restoration of RNA homeostatic mechanisms compromised by tau or represent other mechanisms that have not been previously described. On the other hand, TRA2B is essential for maintaining neuronal viability; its deficiency has been directly linked to massive apoptosis and abnormal cortical proliferation in murine models [81]. Furthermore, TRA2B acts as a direct modulator of MAPT exon 10 splicing, promoting the physiological balance of 4R-tau isoforms [82]. Since local protein synthesis and correct splicing at the synapse are prerequisites for memory consolidation [83], the recovery of this machinery in males may better support the metabolic demands of synaptic repair following tau oligomer clearance. Collectively, these proteomic findings provide a systems-level explanation for the sex-specific therapeutic responses observed in this study.

To fully contextualize these divergent outcomes, the mechanistic basis for this profound sexual dimorphism must be considered. First, prior pharmacokinetic evaluation of SAFit2 has focused solely on young, male mice [24, 49]. Our findings reveal major differences in SAFit2 uptake and distribution by sex, likely driven by metabolic or body composition differences. These sex-dependent pharmacokinetics (e.g., distinct brain-to-plasma ratios) may intrinsically alter the magnitude of target engagement within the CNS, even though overall brain concentrations of SAFit2 (~ 30 nM) remained five-fold higher than the inhibition constant (Ki) for FKBP51 (~ 6 nM) in both sexes. Second, as previously noted, the baseline pathological state diverges by sex; the accelerated tau trajectory in males presents a distinct baseline landscape of FKBP51 availability and tau conformers compared to females. This divergent baseline dictates which specific pathological pools are most receptive to clearance at the time of intervention. Finally, this dimorphism in tau processing is likely heavily influenced by the intricate interplay between FKBP51, glucocorticoid receptor (GR) signaling, and sex hormones. FKBP51 is a documented co-chaperone for the GR, and GR signaling functionally interacts with estrogen receptors (ER) to modulate transcriptional outcomes [84, 85]. Together, these data suggest that the sex-dependent effects of SAFit2 arise not from a fundamentally different target, but from distinct pathological and hormonal contexts that shape how FKBP51 inhibition is translated into tau clearance and proteomic remodeling.

### Limitations

Some of the limitations to consider in the present work include that the PS19 model exhibits an inherent sexual dimorphism in the onset and severity of tau pathology with male mice showing more rapid and consistent pathology [86, 87], which complicates the complete dissection of sex-specific pharmacological responses from baseline phenotypic differences. Also, the intervention started at a symptomatic stage (7.5 months) with a treatment duration of 28 days; while this design highlights the therapeutic potential of targeting FKBP51 in advanced disease, a less intrusive delivery method for the drug or earlier initiation may be required to impact stable aggregates, like those detected by AT8. Another limitation is that due to tissue availability constraints, unbiased proteomic profiling was conducted in the hippocampus, whereas biochemical validation via western blotting utilized cortical homogenates. Although tau pathology is widespread in the PS19 brain, the outcomes could be different due to regional divergence. Finally, the pharmacokinetic profile of SAFit2 is characterized by a relatively short half-life. Consequently, the dosing strategy may have resulted in intermittent rather than continuous target coverage and added stress on the animals from multiple daily injections, suggesting that optimized formulations or more specific ways to modulate FKBP51 like proteolysis targeting chimera (PROTAC) [88], protein-protein disruptors, like LA1011 [73], or the use of antisense oligonucleotides (ASOs) could yield even more robust effects in future studies.

## Conclusions

Together, these findings suggest FKBP51 inhibition as a promising therapeutic strategy for tauopathy by selectively targeting pathogenic tau species and reshaping disease-relevant proteomic networks in a sex-dependent manner.

## Supplementary Information

Supplementary Material 1.

Supplementary Material 2.

## Data Availability

Data is provided within the manuscript or supplementary information files. The mass spectrometric data were deposited to Mass Spectrometry Interactive Virtual Environment (MassIVE), which is part of the ProteomeXchange Consortium, with the dataset identifier MSV000100258.

## Abbreviations

AD: Alzheimer’s Disease
ANOVA: Analysis of Variance
ASO: Antisense Oligonucleotide
AT8: Antibody recognizing phosphorylated Tau at Ser202/Thr205
BBB: Blood–Brain Barrier
BCA: Bicinchoninic Acid (Protein Assay)
CAMK2B: Calcium/Calmodulin–Dependent Protein Kinase II Beta
CORT: Corticosterone
Cx: Cortex
DAA: Differential Abundance Analysis
DEP: Differentially Expressed Protein
DAPI: 4′,6–Diamidino–2–Phenylindole (Nuclear Stain)
DG: Dentate Gyrus
ECx: Entorhinal Cortex
ELISA: Enzyme–Linked Immunosorbent Assay
FDR: False Discovery Rate
FKBP51: FK506–Binding Protein 51
GFAP: Glial Fibrillary Acidic Protein
GO: Gene Ontology
HMW: High Molecular Weight
HPA: Hypothalamic–Pituitary–Adrenal (Axis)
HPCA: Hippocalcin
hnRNP: Heterogeneous Nuclear Ribonucleoprotein
i.p.: Intraperitoneal (Injection)
IACUC: Institutional Animal Care and Use Committee
Iba: Ionized Calcium–Binding Adapter Molecule 1 (Microglial Marker)
IF: Immunofluorescence
Ki: Inhibition Constant
LC: MS/MS QqQ–Liquid Chromatography–Tandem Mass Spectrometry Triple Quadrupole
LFQ: Label–Free Quantification
MBprot: Mode Between Protein (Normalization)
MCODE: Molecular Complex Detection (Network Analysis Algorithm)
MS: Mass Spectrometry
MSqRob: Mass Spectrometry Quantification Robust Ridge Regression
NOR: Novel Object Recognition (Test)
NTg: Non–Transgenic (Mice)
PBS: Phosphate–Buffered Saline
PCx: Posterior Cortex
PFA: Paraformaldehyde
PPI: Protein–Protein Interaction
PROTAC: PROteolysis TArgeting Chimera
PS19: P301S Tau Transgenic Mouse Model
RAWM: Radial Arm Water Maze
RNA: Ribonucleic Acid
SART1: Spliceosome Associated Factor 1
SEM: Standard Error of the Mean
SDS: Sodium Dodecyl Sulfate
TST: Tail Suspension Test
TOC1: Antibody recognizing oligomeric tau
TRA2B: Transformer 2 Beta Homolog (Splicing Factor)
VSN: Variance Stabilizing Normalization
WB: Western Blot
