# Supplementary material for: FKBP51 inhibition by SAFit2 modulates tau pathology and cognitive deficits in PS19 mice
Source: Alzheimers Res Ther. 2026 Jun 6;18:183. doi: 10.1186/s13195-026-02107-3 (PMC13463937; doi:10.1186/s13195-026-02107-3)
Supplement: Supplementary file 1 [file 13195_2026_2107_MOESM1_ESM.docx]

**Supplementary Materials**

**Supplementary Fig. 1 Extended behavioral characterization and validation controls for SAFit2-treated mice. (a-b)** Open Field test parameters showing no significant differences in **(a)** mean speed (m/s) and **(b)** time spent in the center (% of total time) across groups, during the 10min test. **(c)** Quantification of the total exploration time (seconds) during the Novel Object Recognition (NOR) test phase for NTg and PS19 mice. The dotted horizontal line indicates the exclusion criterion of 10 s; animals falling below this threshold were excluded from the main analysis (Fig. 3a) to ensure valid sampling. **(d)** NOR Discrimination Index including the complete cohort of tested animals (without applying the 10 s minimum exploration exclusion threshold). This inclusive analysis confirms that the presence of low-exploring mice does not significantly skew the overall behavioral trends or effect sizes. **(e)** Latency to first immobility (seconds) in the Tail Suspension Test (TST), demonstrating that the initial motivational drive to escape remains intact and is unaltered by SAFit2 treatment. Statistical analyses were performed using three-way ANOVA (Genotype x Treatment x Sex) followed by Bonferroni’s multiple comparisons test. Non-normally distributed datasets were analyzed using the non-parametric Kruskal-Wallis test. Data are presented as mean ± SEM with individual data points. Exact final sample sizes (n = 9-13 mice/group) for each experimental group and specific assay are detailed in Supplementary Table 15. Detailed statistics are provided in Supplementary Table 4.

**Supplementary Fig. 2 SAFit2 treatment reduces multimeric AT8 tau in the Anterior Cortex (ACx) of female PS19 mice.** Semi-denaturing western blot analysis distinguishing between physiological monomeric (~68 kDa) and multimeric (≥75 kDa) tau species in ACx homogenates from PS19 mice. Representative blots and quantification of **(a-b)** Total Tau (Tau5), **(c-d)** pT231 Tau, and **(e-f)** AT8 tau are shown. Ratios of pTau to Total Tau (AT8/Tau5 and pT231/Tau5) calculated for the monomeric and the multimeric fraction in **(g)** females and **(h)** males. Protein levels were normalized to stain-free total protein displayed in Supplementary Fig. 4. Data are presented as mean ± SEM. n = 11-12 mice per group. Statistical significance was determined by two-way ANOVA (Sex × Treatment) followed by Bonferroni’s multiple comparisons test. Non-normally distributed datasets were analyzed using the Kruskal-Wallis test followed by Dunn’s multiple comparisons test. *p < 0.05 vs. vehicle. Detailed statistics are provided in Supplementary Table 5.

**Supplementary Fig. 3. Biochemical profiling of monomeric and multimeric tau in the hippocampus (HPC).** Semi-denaturing western blot analysis distinguishing between physiological monomeric (~68 kDa) and multimeric (≥75 kDa) tau species in HPC homogenates from PS19 mice. Representative blots and quantification of **(a-b)** Total Tau (Tau5), **(c-d)** pT231 Tau, and **(e-f)** AT8 tau are shown. Ratios of pTau to Total Tau (AT8/Tau5 and pT231/Tau5) calculated for the monomeric and the multimeric fraction in **(g)** females and **(h)** males. Protein levels were normalized to stain-free total protein displayed in Supplementary Fig. 4. Data are presented as mean ± SEM. n = 10-12 mice per group. Statistical significance was determined by two-way ANOVA (Sex × Treatment) followed by Bonferroni’s multiple comparisons test. Non-normally distributed datasets were analyzed using the Kruskal-Wallis test followed by Dunn’s multiple comparisons test. **p < 0.01 vs. vehicle. Detailed statistics are provided in Supplementary Table 7.

**Supplementary Fig. 4 Stain-free total protein loading controls for semi-denaturing western blots.** Stain-free total protein gels were used as the loading controls for semi-denaturing western blot quantifications. Representative blots are shown corresponding to the analysis of the Anterior Cortex (ACx) for **(a)** Tau5, **(b)** pT231 tau, and **(c)** AT8; the Posterior Cortex (PCx) for **(d)** Tau5, **(e)** pT231 tau, and **(f)** AT8; and the Hippocampus (HPC) for **(g)** Tau5, **(h)** pT231 tau, and **(i)** AT8. Molecular weight markers are indicated in kDa on the left.

**Supplementary Fig. 5 Basal monomeric tau remains unaltered by SAFit2 under fully denaturing conditions in the Posterior Cortex (PCx).** Fully denaturing western blot analysis (boiled lysates) validating antibody specificity and assessing the monomeric tau pool in PCx homogenates from PS19 mice. (a) Representative blots demonstrating the detection of specific monomeric bands (68 kDa) for Total Tau (Tau5), AT8, and pT231 tau. Actin was used as a loading control. Quantification of absolute protein levels for (b) Tau5, (c) AT8, and (d) pT231 tau normalized to Actin. Ratios of phosphorylated tau to total tau are shown for (e) AT8/Tau5 and (f) pT231/Tau5. Data are presented as mean ± SEM. n = 11-12 mice per group. Statistical significance was determined by two-way ANOVA (Sex × Treatment) followed by Bonferroni’s multiple comparisons test. *p < 0.05 vs. vehicle. Detailed statistics are provided in Supplementary Table 7.

**Supplementary Fig. 6 Impact of chronic SAFit2 treatment on Total Tau burden in the hippocampus and cortex.** Quantitative analysis and representative images of Total Tau immunoreactivity (red) and DAPI (blue) of **(a-b)** female and **(c-d)** male PS19 mice treated with vehicle (red bars) or SAFit2 (blue bars). Analysis includes prefrontal cortex (Cx), dorsal and ventral hippocampal subfields (dCA1, dCA3, dDG, vCA1, vCA3, vDG), and enthorinal cortex (ECx) regions. Data are presented as mean ± SEM with individual data points (n = 10-12 mice/group). Statistical significance was determined by two-way ANOVA (Sex × Treatment) followed by Bonferroni’s multiple comparisons test. Non-normally distributed datasets were analyzed using the Kruskal-Wallis test followed by Dunn’s multiple comparisons test. *p < 0.05, scale bars: 50 µm. Detailed statistics are provided in Supplementary Table 7.

**Supplementary Fig. 7 Region-specific modulation of pT231 phosphorylation following SAFit2 treatment.** Quantitative analysis of pT231 immunoreactivity (red) and representative figures from the hippocampus and cortex of **(a-b)** female and **(c-d)** male PS19 mice. Analysis includes the prefrontal cortex (Cx), hippocampal subfields (dCA1, dCA3, dDG, vCA1, vCA3 and vDG) and enthorinal cortex (ECx) of SAFit2-treated (blue bars) compared to vehicle-treated (red bars) controls. Data are presented as mean ± SEM with individual data points (n = 10-12 mice/group). Statistical significance was determined by two-way ANOVA (Sex × Treatment) followed by Bonferroni’s multiple comparisons test. Non-normally distributed datasets were analyzed using the Kruskal-Wallis test followed by Dunn’s multiple comparisons test. *p < 0.05, scale bars: 50 µm. Detailed statistics are provided in Supplementary Table 8.

**Supplementary Fig. 8 Validation of pT231 antibody specificity and staining patterns.** Validation of pT231 antibody using negative and positive biological controls in the **(a)** cortex and **(b)** hippocampus. Representative immunofluorescence images of pT231 (red) and DAPI (blue) staining in tissues from Tau Knockout (Tau KO) – negative control, hTau mice (expressing all 6 isoforms of human tau and lacking mouse tau) - positive control, as well as representative images of experimental groups from this study (Female/Male NTg and PS19) treated with Vehicle or SAFit2. Scale bars: 50 µm.

**Supplementary Fig. 9 Spatial distribution of AT8 and TOC1 tau pathology in the dorsal hippocampus and entorhinal cortex.** Representative immunofluorescence micrographs showing the localization of **(a)** AT8-positive tau (red) and **(b)** oligomeric tau species (TOC1, red) in the dorsal hippocampal subfields (dCA1, dCA3, dDG), and entorhinal cortex (ECx) of female and male PS19 mice treated with SAFit2 or vehicle. DAPI nuclei stain is shown in blue. Scale bars: 50 µm. These images expand on the regions displayed in Fig. 5 and Fig. 6, respectively.

**Supplementary Fig. 10 Sex-specific modulation of astrocytic gliosis (GFAP) in PS19 mice.** Quantitative analysis and representative images of GFAP immunoreactivity (green) in the hippocampus and cortex of **(a)** female and **(b)** male NTg and PS19 mice. Analysis was performed in different brain regions including prefrontal cortex (Cx), dorsal and ventral hippocampus subfields (dCA1, dCA3, dDG, vCA1, vCA3 and vDG), and entorhinal cortex (ECx) comparing SAFit2-treated (orange and blue bars) to vehicle-treated (black and red bars) controls. Nuclei are counterstained with DAPI (blue). Data are presented as mean ± SEM (n = 10-12 mice/group). Statistical significance was determined by three-way ANOVA (Sex × Treatment × Genotype) followed by Bonferroni’s multiple comparisons test. Non-normally distributed datasets were analyzed using the Kruskal-Wallis test followed by Dunn’s multiple comparisons test. *p < 0.05, **p < 0.01, ***p < 0.001, ****p < 0.0001. Scale bars: 50 µm. Detailed statistics are provided in Supplementary Table 12.

**Supplementary Fig. 11 SAFit2 treatment does not alter microglial activation in PS19 mice.** Quantitative analysis and representative images of Iba-1 immunoreactivity (yellow) in the hippocampus and cortex of **(a, b)** female and **(c, d)** male NTg and PS19 mice. Analysis was performed in different brain regions including prefrontal cortex (Cx), dorsal and ventral hippocampus subfields (dCA1, dCA3, dDG, vCA1, vCA3 and vDG), and entorhinal cortex (ECx) comparing SAFit2-treated (orange and blue bars) to vehicle-treated (black and red bars) controls. Nuclei are counterstained with DAPI (blue). Data are presented as mean ± SEM (n = 10-12 mice/group). Statistical significance was determined by three-way ANOVA (Sex × Treatment × Genotype) followed by Bonferroni’s multiple comparisons test. Non-normally distributed datasets were analyzed using the Kruskal-Wallis test followed by Dunn’s multiple comparisons test. *p < 0.05, **p < 0.01, ***p < 0.001, scale bars: 50 µm. Detailed statistics are provided in Supplementary Table 13.

**Supplementary Fig. 12 Spatial distribution of FKBP51 in the hippocampus and entorhinal cortex.** Representative immunofluorescence micrographs showing the localization of FKBP51 in **(a)** female and **(b)** male NTg and PS19 mice treated. Images show the comparison of SAFit2-treated and vehicle-treated controls in the dorsal and ventral hippocampal subfields (dCA3, dDG, vCA1, vCA3 and vDG), and entorhinal cortex (ECx). DAPI nuclei stain is shown in blue. Scale bars: 50 µm. These images expand on the regions displayed in Fig. 7**.**

**Supplementary Fig. 13 Heatmap of SAFit2 GO terms across groups.** Heatmap of hierarchically clustered GO terms (GO Biological Process, GO Molecular Function, and GO Cellular Component) enrichment based on the differentially expressed proteins (DEPs) based on SAFit2 treatment in NTg and PS19 male and female mice. Note: no DEPs were identified based on treatment in male NTg mice. Color intensity represents statistical significance (-log10 P-value) with white denoting absence of enrichment.
